# Girard Derivatization-Based Enrichment Strategy for Profiling the Carbonyl Submetabolome in Biological Samples

**DOI:** 10.1021/acs.analchem.4c04037

**Published:** 2024-12-19

**Authors:** Xin Tao, Jia-Yue Liu, Jun-Yi Zhou, Jiang-Kun Dai, Zeyu Xiao, Hou-Kai Li, Jian-Bo Wan

**Affiliations:** † State Key Laboratory of Quality Research in Chinese Medicine, Institute of Chinese Medical Sciences, University of Macau, Taipa, Macao SAR 999078, China; ‡ Collaborative Translational Medicine Collaborative Innovation Center, Department of Pharmacology and Chemical Biology, Institute of Medical Sciences, Shanghai Jiao Tong University School of Medicine, Shanghai 200025, China; § School of Pharmacy, Shanghai University of Traditional Chinese Medicine, Shanghai 201203, China

## Abstract

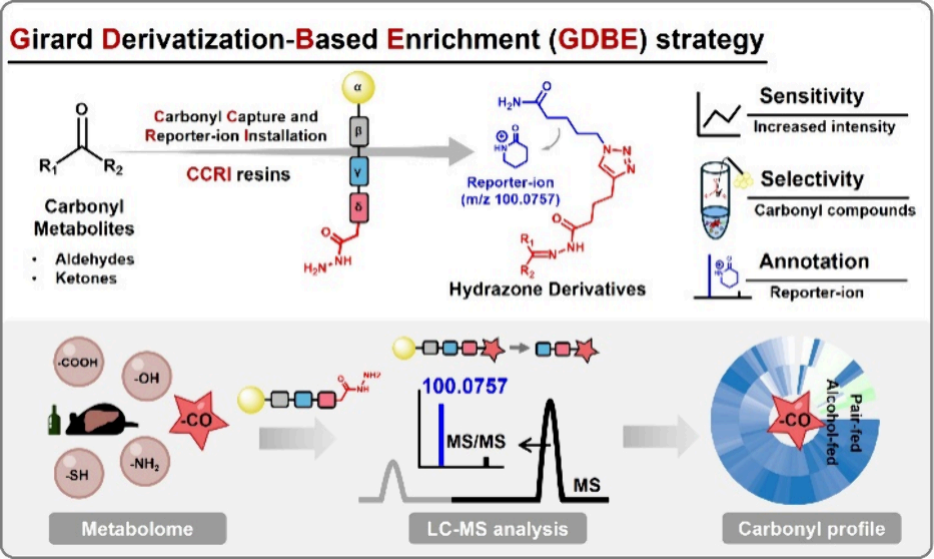

Numerous bioactive compounds containing carbonyl groups, including aldehydes and ketones, are widely acknowledged as potential biomarkers for several diseases and are implicated in the development of metabolic disorders. However, the detection of carbonyl metabolites is hindered by challenges, such as poor ionization efficiency, low biological concentration, instability, and complexity of the sample matrix. To overcome these limitations, we developed a Girard derivatization-based enrichment (GDBE) strategy for capturing and comprehensively profiling carbonyl metabolites in biological samples. A functionalized resin, named carbonyl capture and reporter-ion installation (CCRI) resins, was synthesized to selectively capture carbonyl metabolites via a Girard reaction. After unwanted metabolites were removed, the hydrazone derivatives were cleaved from the solid-phase resins and subjected to LC-MS analysis. The proposed GDBE strategy exhibits exceptional selectivity for capturing and enriching carbonyl metabolites. Moreover, this method surpasses current detection limits by enhancing the MS sensitivity and facilitating structural characterization of hydrazone derivatives by a specific MS/MS fragmentation signature. Using the GDBE method, 957 potential carbonyl metabolites were successfully identified in liver tissue from alcohol-fed mice. Among them, 76 carbonyl metabolites were annotated, indicating the potential of this strategy for the efficient nontargeted profiling of the carbonyl submetabolome in complex biological samples.

Endogenous carbonyl metabolites, including aldehydes and ketones, are naturally occurring molecules, comprising a large proportion of the human metabolome. These reactive molecules are generally produced through various metabolic processes, including peroxidation, inflammation, ketogenesis, and carbohydrate metabolism.^1^ Reactive oxygen species (ROS), including superoxide, hydroxyl radical, and hydrogen peroxide, stimulate lipid peroxidation of unsaturated fatty acids, especially n-3 and n-6 fatty acids, to form carbonyl molecules. These highly reactive carbonyl molecules cause oxidative modification of cellular macromolecules, resulting in DNA damage, protein denaturation, and further lipid peroxidation of the cellular membrane.^2,3^ Furthermore, serving as the signaling molecules, endogenous carbonyl steroids play a crucial regulatory role in growth, development, reproduction, and the maintenance of environmental homeostasis in the human body.^4,5^ Therefore, carbonyl compounds are closely implicated in the occurrence and progression of several diseases and have been considered as the potential molecular signatures for oxidative stress and various diseases, including metabolic diseases,^6^ neurodegenerative disease^7^ and cancers.^8,9^ Thus, the comprehensive profiling of carbonyl metabolites is imperative to understand their roles in physiological and pathological processes.

Due to instability, poor ionization properties, and low abundance,^1,2,10,11^ the detection of carbonyl molecules in biological samples by liquid chromatography coupled with mass spectrometry (LC-MS) remains a challenge. The chemical labeling strategy followed by LC-MS has been extensively used for the analysis of carbonyl molecules in biological samples. The carbonyl group of metabolites was specifically reacted with several derivatization reagents, such as alkoxyamines,^1^ hydrazines,^2^ and 4-(2-and (trimethylammonio) ethoxy) benzenaminium halide (4-APC).^12^ By incorporating a functional group with a permanent charge or other groups that enhanced ionization efficiency, chemical labeling strategy greatly improves MS sensitivity and fragmentation characteristics and chromatographic performance as well. However, chemical derivatization, followed by LC-MS, might be generally limited by interference from the complicated sample matrix and intrinsic metabolites, resulting in ion suppression. To overcome these restraints, in recent years, several functional resins^13^ and magnetic beads^1,14^ with an aminooxy moiety were developed to chemoselectively capture carbonyl molecules for accurate quantification^13^ and comprehensive profiling.^1,14^ These chemoselective strategies allow separation of the captured metabolites from the sample matrix, facilitating targeted discovery of unknown carbonyl metabolites in biological samples. In this study, a Girard derivatization-based enrichment (GDBE) strategy was developed to selectively capture and comprehensively profile carbonyl metabolites in biological samples. This strategy was accomplished by a functional resin with a hydrazide moiety that selectively captures carbonyl metabolites via a Girard reaction under mild conditions. After undesired metabolites were removed, the hydrazone derivatives were cleaved from the solid-phase resins and subjected to LC-MS analysis.

## Materials and Methods

### Chemicals and Materials

Twenty carbonyl standards (Table 1), including 16 aldehydes and 4 ketones, were procured from Sigma-Aldrich (St. Louis, MO, U.S.A.), Aladdin (Shanghai, China), TCI (Shanghai, China), Sangon (Shanghai, China), and J&K Scientific (Beijing, China). Their chemical structures are depicted in Figure S1. NovaPEG Rink Amide resins were obtained from Sigma-Aldrich (St. Louis, MO, U.S.A.). 5-Azidopentanoic acid was sourced from Cayman Chemical Company (Ann Arbor, MI, U.S.A.), while alkyne hydrazide was purchased from Lumiprobe Corporation (Hunt Valley, MD, U.S.A.). 2-(1*H*-Benzotriazole-1-yl)-1,1,3,3-tetramethylammonium tetrafluoroborate (TBTU) and *N*,*N*-diisopropylethylamine (DIEA) were acquired from Aladdin (Shanghai, China). *N*,*N*-Dimethylformamide (DMF) and trifluoroacetic acid (TFA) were obtained from Macklin (Shanghai, China). 2-(4-((Bis((1-(*tert*-butyl)-1*H*-1,2,3-triazol-4-yl)methyl)amino)methyl)-1*H*-1,2,3-triazol-1-yl) acetic acid (BTTAA) and triisopropyl silane (TIPS) were procured from Energy Chemical (Shanghai, China). *N*-Methyl-2-pyrrolidone (NMP) was procured from Adamas (Shanghai, China).

**Table 1:** Detailed Information on 20 Carbonyl Standards and Their Hydrazone Derivatives^a^

			before GDBE (native carbonyls)				after GDBE (hydrazone derivatives)				
No.	carbonyl standards	type	formula	M.W.	RT (min)	LOD (nM)	formula	observed *m*/*z*	RT (min)	LOD (nM)	fold of LOD decrease
**1**	galacturonic acid	aldehydes	C_6_H_10_O_7_	194.0427	0.56	100	C_17_H_28_N_6_O_8_	445.2065	0.69	10.00	10
**2**	α-ketoglutaric acid	ketones	C_5_H_6_O_5_	146.0215	0.92	100	C_16_H_24_N_6_O_6_	397.1813	5.63	1.00	100
**3**	vanillin	aldehydes	C_8_H_8_O_3_	152.0473	6.65	250	C_19_H_26_N_6_O_4_	403.2055	6.73	0.10	2500
**4**	4-formylbenzoic acid	aldehydes	C_8_H_6_O_3_	150.0317	6.78	100	C_19_H_24_N_6_O_4_	401.1924	6.86	0.01	10000
**5**	pentanal	aldehydes	C_5_H_10_O	86.0732	-	-	C_16_H_28_N_6_O_2_	337.2339	7.66	0.01	
**6**	benzaldehyde	aldehydes	C_7_H_6_O	106.0419	-	-	C_18_H_24_N_6_O_2_	357.2050	7.84	0.003	
**7**	phenylacetaldehyde	aldehydes	C_8_H_8_O	120.0575	-	-	C_19_H_26_N_6_O_2_	371.2221	8.02	10.00	
**8**	hexanal	aldehydes	C_6_H_12_O	100.0888	-	-	C_17_H_30_N_6_O_2_	351.2498	8.71	0.01	
**9**	4-hydroxynonenal	aldehydes	C_9_H_16_O_2_	156.1150	-	-	C_20_H_34_N_6_O_3_	407.2732	8.89	0.01	
**10**	cinnamaldehyde	aldehydes	C_9_H_8_O	132.0575	-	-	C_20_H_26_N_6_O_2_	383.2158	8.94	0.10	
**11**	heptanal	aldehydes	C_7_H_14_O	114.1045	-	-	C_18_H_32_N_6_O_2_	365.2675	9.74	0.25	
**12**	dehydroandrosterone	ketones	C_19_H_28_O_2_	288.2089	12.60	100	C_30_H_46_N_6_O_3_	539.3655	9.95	10.00	10
**13**	benzophenone	ketones	C_13_H_10_O	182.0732	13.44	250	C_24_H_28_N_6_O_2_	433.2339	10.67	50.00	5
**14**	octanal	aldehydes	C_8_H_16_O	128.1201	-	-	C_19_H_34_N_6_O_2_	379.2789	10.74	0.02	
**15**	nonanal	aldehydes	C_9_H_18_O	142.1358	-	-	C_20_H_36_N_6_O_2_	393.2943	11.67	0.003	
**16**	progesterone	ketones	C_21_H_30_O_2_	314.2246	15.06	5	C_32_H_48_N_6_O_3_	565.3865	11.98	0.25	20
**17**	decanal	aldehydes	C_10_H_20_O	156.1514	-	-	C_21_H_38_N_6_O_2_	407.3084	12.60	0.01	
**18**	undecanal	aldehydes	C_11_H_22_O	170.1671	-	-	C_22_H_40_N_6_O_2_	421.3260	13.52	0.01	
**19**	dodecanal	aldehydes	C_12_H_24_O	184.1827	-	-	C_23_H_42_N_6_O_2_	435.3440	14.45	0.003	
**20**	hexadecenal	aldehydes	C_16_H_30_O	238.2297	-	-	C_27_H_48_N_6_O_2_	489.3942	17.97	0.001	

a RT, retention time; -, undetectable; LOD, limit of detection.

### Synthesis of Carbonyl Capture and Reporter-Ion Installation (CCRI) Resins

NovaPEG Rink Amide resins (Novabiochem, Sigma-Aldrich), with a loading capacity of 0.35 mmol/g (50 mg, 0.0175 mmol, 1 equiv), were placed in a dried vial with 3 mL of dry DMF. Then, 5-azidopentanoic acid (3.8 mg, 0.027 mmol, 1.5 equiv), TBTU (16.9 mg, 0.053 mmol, 3 equiv), and DIEA (9 μL, 0.053 mmol, 3 equiv) were sequentially added to the reaction mixture. The vial was then placed on a magnetic stirrer at room temperature for 10 h. A Kaiser test was used to examine the completion of the acylation reaction. Subsequently, the reaction mixture was washed with NMP (5 mL), water (5 mL) and NMP (5 mL). After dryness under a nitrogen stream, the azide-modified resins were dissolved in NMP at a concentration of 20 mg/mL and stored at 4 °C. In the second step, CCRI resin was synthesized by click chemistry.^15^ The synthesized azide-modified resins (100 μL, 20 mg/mL) were added to 280 μL of click reaction cocktail (100 μL of 20 mM alkyne hydrazide, 15 μL of 50 mM CuSO_4_, 15 μL of 200 mM BTTAA, and 50 μL of 1 M ascorbic acid, dissolved in H_2_O). The reactions were conducted by incubating the mixture on a ThermoMixer at 1000 rpm and 30 °C for 6 h. After the centrifugation at 10000 rpm for 3 min, CCRI resin was pelleted and consecutively washed with DMF/water (1:3, 500 μL), DMF/water (1:1, 500 μL), and DMF (500 μL). The resin was then evaporated to dryness under a nitrogen stream and stored at 4 °C.

### Animals and Treatments

Male C57BL/6 mice were procured from Vital River Laboratory Animal Technology Co., Ltd. (Beijing, China) and housed in a controlled specific-pathogen-free (SPF) environment. To induce alcoholic liver injury, we utilized the well-established National Institute on Alcohol Abuse and Alcoholism (NIAAA) animal model of chronic-plus-binge alcohol feeding, as previously described.^16−19^ After 5 days of acclimatization to a liquid control diet (ROPHIC Animal Feed High-Tech Co., Ltd., Jiangsu, China) provided *ad libitum*, the mice were randomly assigned to pair-fed and alcohol-fed groups. The pair-fed group was fed a Lieber-DeCarli ethanol (5%, *v*/*v*) liquid diet for 10 days, while the pair-fed group received a control liquid diet containing isocaloric maltose dextrin. On the 16th day, the pair-fed and alcohol-fed mice were gavaged with a single dose of ethanol (5 g/kg) and an isocaloric maltose dextrin solution, respectively. After a 9h fasting, all mice were anesthetized using 1% pentobarbital sodium, and liver tissue samples were harvested for subsequent analysis. Prior approval of animal protocols was obtained from the Institutional Animal Ethics Committee of the Shanghai University of Traditional Chinese Medicine (PZSHUTCM210115004).

### Girard Derivatization-Based Enrichment

Liver tissue from the same lobe (50 mg) was ground in 80% MeOH (500 μL) and then centrifuged at 12000 rpm for 10 min at 4 °C.^11^ The collected supernatant or carbonyl standard mixture was mixed with CCRI resins (2 mg) and incubated at 50 °C for 2 h for the Girard reaction. The carbonyl-bound resins were successively washed with DMF/water (1:3, 500 μL), DMF/water (1:1, 500 μL) and DMF (500 μL), and evaporated to dryness under a nitrogen stream. TFA (100 μL) was used to cleave carbonyl metabolites from the resins, and the cleavage reaction was performed at 1,000 rpm and 20 °C for 15 min twice, the supernatant was combined into a fresh tube and evaporated under a nitrogen stream. The residue was dissolved in 100 μL of ACN and centrifuged at 14800 rpm for 10 min before LC-MS analysis.

### LC-MS Analysis

LC-MS analysis was conducted using a Waters ACQUITY UPLC instrument coupled with a SYNAPT G2-Si Q-TOF high-resolution mass spectrometer (Waters Corp., Manchester, U.K.). Chromatographic separation was achieved on an ACQUITY UPLC HSS T3 column (100 mm × 2.1 mm, 1.8 μm) at a controlled temperature of 30 °C. The binary mobile phase consisting of aqueous formic acid solution (0.1%, *v*/*v*, Solvent A) and acetonitrile containing 0.1% formic acid (*v*/*v*, Solvent B) was used at a flow rate of 0.5 mL/min. The gradient started at 1% B for 2 min after injection and increased linearly to 100% B at 23 min. The system was maintained at 100% B for 4 min before returning to 1% B. The sample injection volume and autosampler temperature were set at 3 μL and 10 °C, respectively. The LC eluent was introduced to a SYNAPT G2-Si MS instrument equipped with an electrospray ionization source (ESI). The Q-TOF MS was operated in the positive ion mode, with the following key parameters: capillary voltage of 3.0 kV, sample cone voltage of 40 V, source temperature of 100 °C, desolvation temperature of 250 °C, nitrogen gas flow of 600 L/h, cone gas flow of 50 L/h, and TOF acquisition rate of 1.5 s/scan. The MS data were collected with a mass range from *m*/*z* 30 to 900 using the MS^E^ Continuum scan mode. The collision energy was operated from 5 to 30 V, and the leucine-enkephalin calibrant solution was continuously infused into the MS during analysis.^20−22^

### Statistical Analysis

Data are presented as means ± SEM. The bar plots were generated with Prism 8.0 software (GraphPad, La Jolla, CA, U.S.A.). The differences between groups were assessed using a student *t*-test; *p* < 0.05 was considered statistically significant. To examine the distributions and discriminations between the alcohol-fed group and pair-fed group of mice based on the carbonyl compounds profile, Orthogonal Partial Least Squares-Discriminant Analysis (OPLS-DA) was conducted using SIMCA-P software (version 14.0, Umetrics, Umeå, Sweden).

## Results and Discussion

### GDBE Strategy

**Figure 1:**
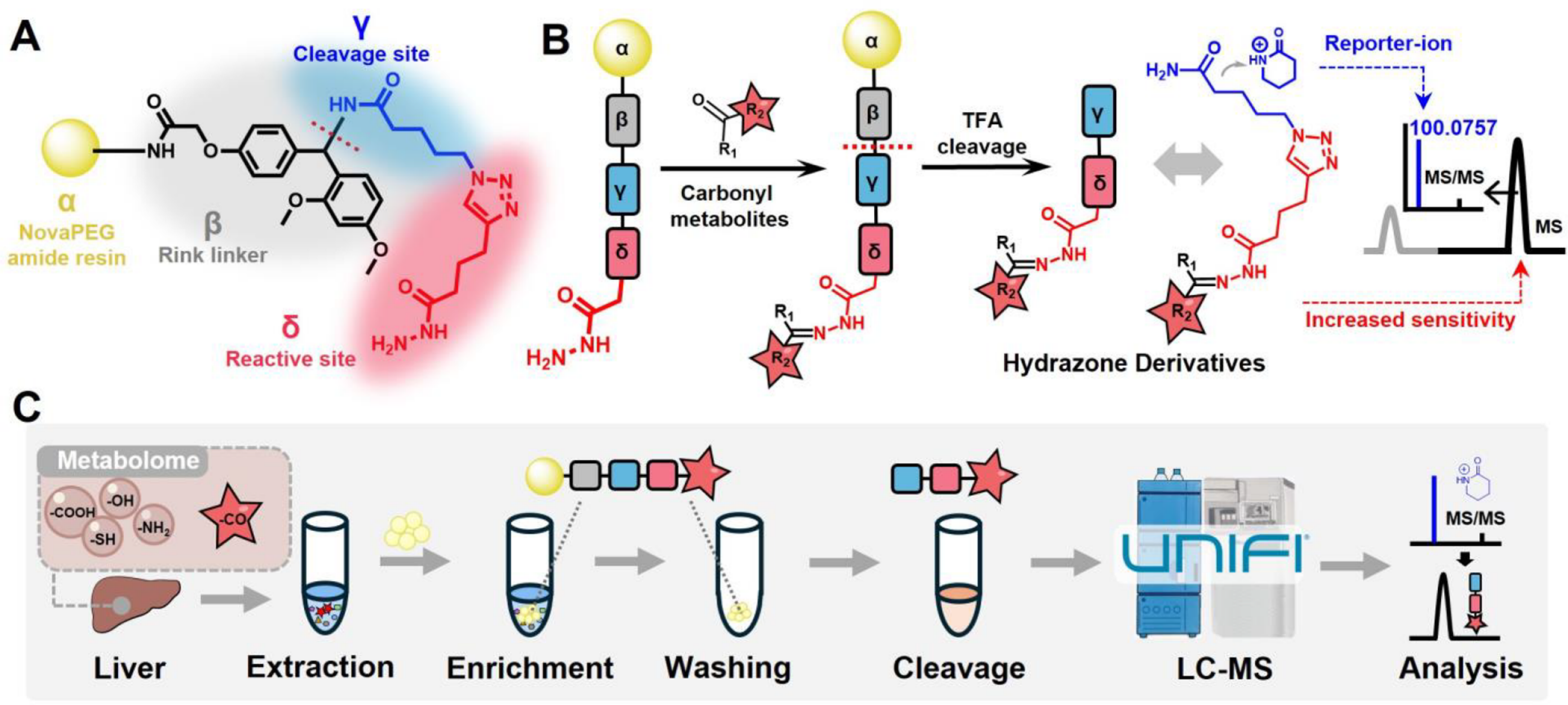
Schematic illustration for GDBE strategy for enrichment and profiling of carbonyl metabolites in biological samples. (A) The scaffold of carbonyl capture and reporter-ion installation (CCRI) resins. The reactive site bearing a hydrazide moiety (δ unit, red) is connected to cleavage site (γ unit, blue), which is immobilized to NovaPEG resins (α unit, yellow) by a modified Rink linker (β unit, black). (B) Schematic overview for enrichment and profiling of carbonyl metabolites using CCRI resins. (C) The general workflow for GDBE strategy.

Comprehensive profiling of carbonyl metabolites in biological samples is imperative for understanding their biochemical activities and roles in physiological and pathological processes. In this study, a GDBE strategy was developed to enrich and profile carbonyl metabolites, including aldehydes and ketones, in biological samples by LC-MS. A functional hydrazide-modified resin, named carbonyl capture and reporter-ion installation (CCRI), was designed and synthesized to chemoselectively capture carbonyl metabolites for enrichment. The scaffold of CCRI resin comprises four key components (Figure 1A), the reactive site bearing a hydrazide moiety (δ unit, red) is connected to the cleavage site (γ unit, blue), which is immobilized to NovaPEG resins (α unit, yellow) by a modified Rink linker (β unit, black). The hydrazide moiety on the CCRI resin allows for capturing carbonyl metabolites in biological samples via a Girard reaction under mild conditions. After the carbonyl-bound resins were washed to remove undesired metabolites, the hydrazone derivatives are cleaved from the solid-phase resins under acidic conditions for LC-MS analysis (Figure 1B). The cleaved hydrazone derivatives consist of two components: carbonyl-reacted δ and γ units. This design provides improved MS detection sensitivity due to the enhanced ionization efficiency of the hydrazone and triazole moieties (δ unit). Additionally, the valeric amide moiety (γ unit) can generate a reporter-ion (*m*/*z* 100.0757) in positive ion mode, offering a characteristic MS/MS fragmentation pattern that facilitates the structural recognition of the hydrazone derivatives derived from carbonyl metabolites (Figure 1B,C).

### Optimization of CCRI Resin Synthesis

The CCRI resin was synthesized through a two-step process involving an acylation reaction, followed by click chemistry. First, azide-modified resins were prepared from NovaPEG Rink Amide resin and 5-azidopentanoic acid through a one-step acylation reaction, as previously described with slight modification.^15,21^ Subsequently, the CCRI resin was synthesized from the azide-modified resins and alkyne hydrazide via a Cu(I)-catalyzed azide–alkyne cycloaddition (CuAAC) reaction, commonly known as click chemistry (Figure S2). To achieve the optimal synthetic efficiency, a series of CuAAC reaction conditions were systematically optimized through a single-factor experiment, including reaction solvents, concentrations of alkyne hydrazide (0.7–21.4 mM), CuSO_4_ (0.7–10.7 mM), ascorbic acid (0.04–0.71 M), and BTTAA (1.3-21.4 mM), reaction temperature (20–60 °C), and duration (1.5–24 h). The acidic cleavage product of CCRI resin (*m*/*z* 269.1721) was monitored by LC-MS. The optimal conditions were determined to be NMP/H_2_O (*v*/*v*, 1:1) as the reaction solution, 7.1 mM alkyne hydrazide, 2.7 mM CuSO_4_, 0.18 M ascorbic acid, 10.7 mM BTTAA, 30 °C reaction temperature, and 6 h reaction duration for the synthesis of CCRI resin (Figure S3).

This immobilized CCRI resin also features a regeneration capability, which can significantly reduce the costs associated with the GDBE strategy. After use, the cleavage product of the CCRI resin can be regenerated through hydrolysis followed by reduction to yield a primary amino group^23^ (Figure S4). Additionally, this enrichment strategy is not limited to capturing carbonyl metabolites alone. For instance, to capture and profile carboxylic and thiol metabolites in biological samples, we need to redesign only the reactive site of resins. Functional resins containing amino and sulfhydryl-reactive chemical groups can be synthesized from azide-modified resins and alkyne propargylamine and *N*-propargylmaleimide via CuAAC reaction (Figure S5). This modified resin allows for the selective capture of carboxylic and thiol metabolites in biological samples, demonstrating the great flexibility and extensibility of this strategy.

### Optimization of GDBE Conditions

Girard’s reagents containing a hydrazide moiety have been widely employed as a derivatization agent with high reactivity to detect carbonyl metabolites in biological samples.^24,25^ In this study, the synthesized CCRI resin also bears a hydrazide group, which may act as a nucleophile to attack the carbonyl groups of aldehydes and ketones under mild conditions, thereby forming stable hydrazone derivatives with easily chargeable tertiary ammonium moieties.^24^ To achieve optimal enrichment performance of GDBE, Girard reaction conditions, including reaction temperature and duration, as well as the cleavage reaction conditions and redissolve solvent, were further optimized using 6 representative aldehyde/ketone standards. As depicted in Figure S6, the signal intensities of the majority of the investigated carbonyl standards reached their maximum values under the following optimized conditions: 50 °C of reaction temperature, 2 h of reaction duration, TFA as the cleavage solvent, 15 min of cleavage duration, and acetonitrile as the redissolve solvent. Furthermore, LC-MS conditions, such as mobile phase modifiers, column temperature, cone voltage, and collision energy, were optimized to achieve the best detection sensitivity, as described in Figure S7. Given the presence of water in biological samples before derivatization, one general approach is to dry the samples and reconstitute them before labeling. To assess the impact of this drying step during sample processing, carbonyl standard solutions were directly incubated with CCRI resin (“– Dry”) or dried and redissolved before incubation with resin (“+ Dry”). A significant decrease in analyte intensity was observed in “+ Dry” group (Figure S8), indicating that sample drying prior to GDBE is unsuitable for accurately detecting carbonyl metabolites, especially volatile small molecule aldehydes and ketones. Our data also demonstrated that the presence of water did not adversely affect Girard’s reaction efficiency, as consistent with previous studies.^11,26^

### Enhancement of Detection Sensitivities of Carbonyl Metabolites after GDBE

**Figure 2:**
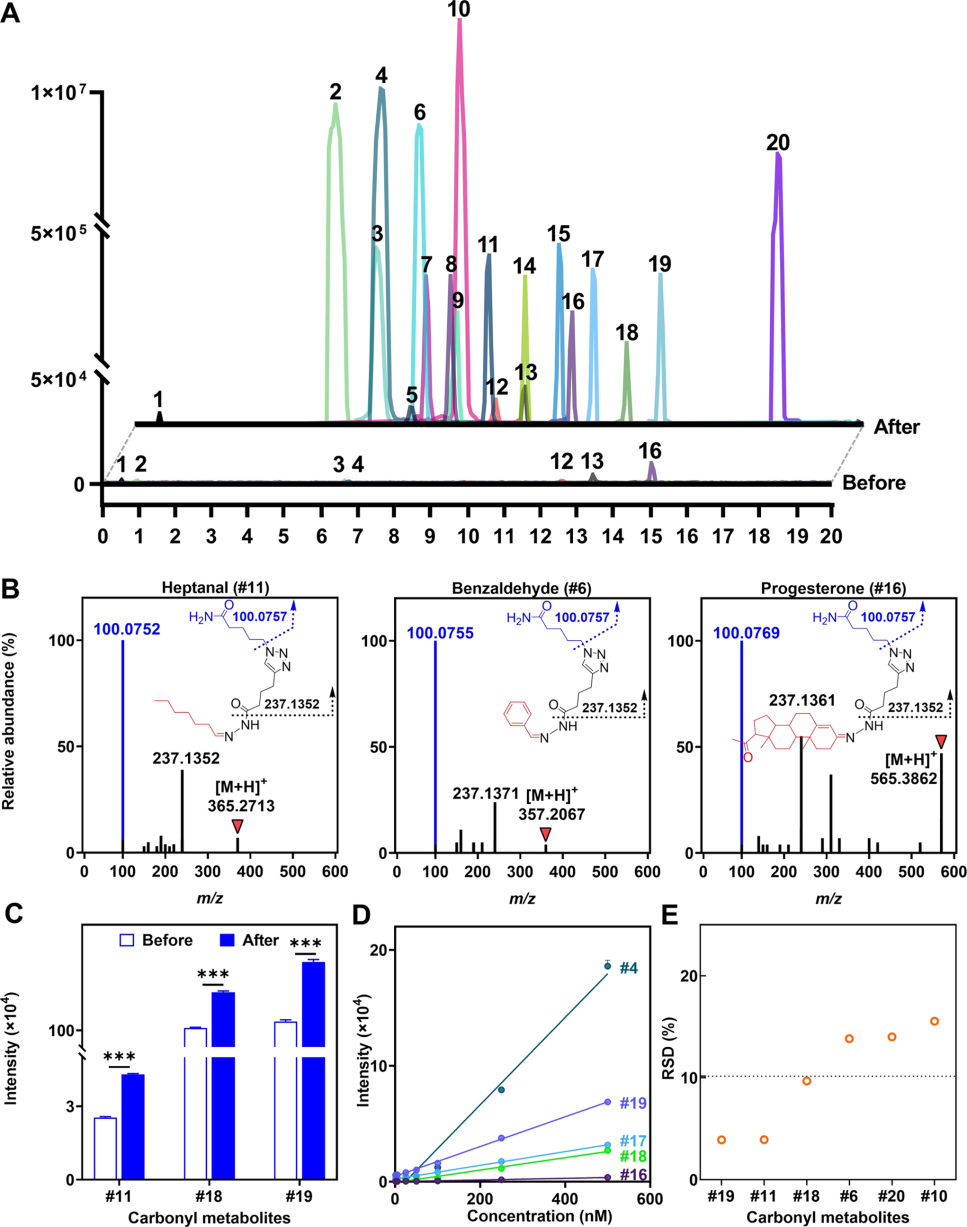
LC-MS characteristics of carbonyl standards using GDBE strategy. (A) Extracted ion chromatograms (EICs) of 20 carbonyl standards before and after GDBE treatment. Carbonyl metabolites after GDBE were detected as their corresponding hydrazone derivatives. (B) MS/MS spectra of hydrazone derivatives derived from representative carbonyl standards. (C) Stability, (D) linearity, and (E) reproducibility of carbonyl metabolites using the GDBE method. The numbers of carbonyl standards are represented in the same manner as in Table 1.

Using the optimized enrichment and detection conditions, 20 representative carbonyl standards (1 μM each), including aliphatic aldehydes, aromatic aldehydes, aliphatic ketones, and aromatic ketones, were analyzed by LC-MS before and after GDBE treatment. Due to their extremely poor ionization property, most aldehyde metabolites could not be detected by LC-MS. Galacturonic acid (#1) and 4-formylbenzoic acid (#4), containing both carboxylic and carbonyl groups, were detectable in negative ion mode. However, after GDBE, all carbonyl metabolites in their hydrazone-derived form were readily detected in positive ion mode (Figure 2A). The GDBE treatment substantially enhanced the detection sensitivities of the carbonyl metabolites, as evidenced by 5- to 10000-fold increase in limits of detection (LODs) (Table 1). The significant increase in detection sensitivities is primarily attributed to the formation of easily ionizable hydrazone derivatives and the presence of the triazole group derived from the resin. Furthermore, the MS/MS spectra of hydrazone derivatives showed a high-intensity diagnostic reporter-ion with an *m*/*z* of 100.0757 and a characteristic fragment with an *m*/*z* of 237.1352 (Figures 2B and S9), facilitating the recognition of carbonyl metabolites. These results indicate that this GDBE method can substantially enhance the MS sensitivity of target carbonyl metabolites and provide a specific MS/MS fragmentation signature.

The stabilities of carbonyl metabolites before and after GDBE treatment were also compared. A freshly prepared carbonyl standard mixture was subjected to GDBE, and the resulting cleaved products were placed at 20 °C for 6 h (“After” group). Another identical standard mixture was placed at 20 °C for 6 h and then subjected to GDBE (“Before” group). As depicted in Figure 2C, the intensities of the three selected carbonyl metabolites in the “After” group were significantly higher than those in the “Before” group, suggesting an increased stability of carbonyl metabolites after GDBE treatment. Furthermore, we observed excellent linear relationships between mass signals and the concentrations of carbonyl metabolites (ranging from 1 to 500 nM) with *R*^2^ values exceeding 0.98 (Figure 2D). The GDBE method also demonstrated good reproducibility with relative standard deviation (RSD) values below 15.5% (Figure 2E). These data indicate that the proposed GDBE approach enables the enrichment of a wide dynamic range of carbonyl metabolites with reliable and reproducible results.

### Influence of TIPS on the Detection of Carbonyl Metabolites Using the GDBE Method

**Figure 3:**
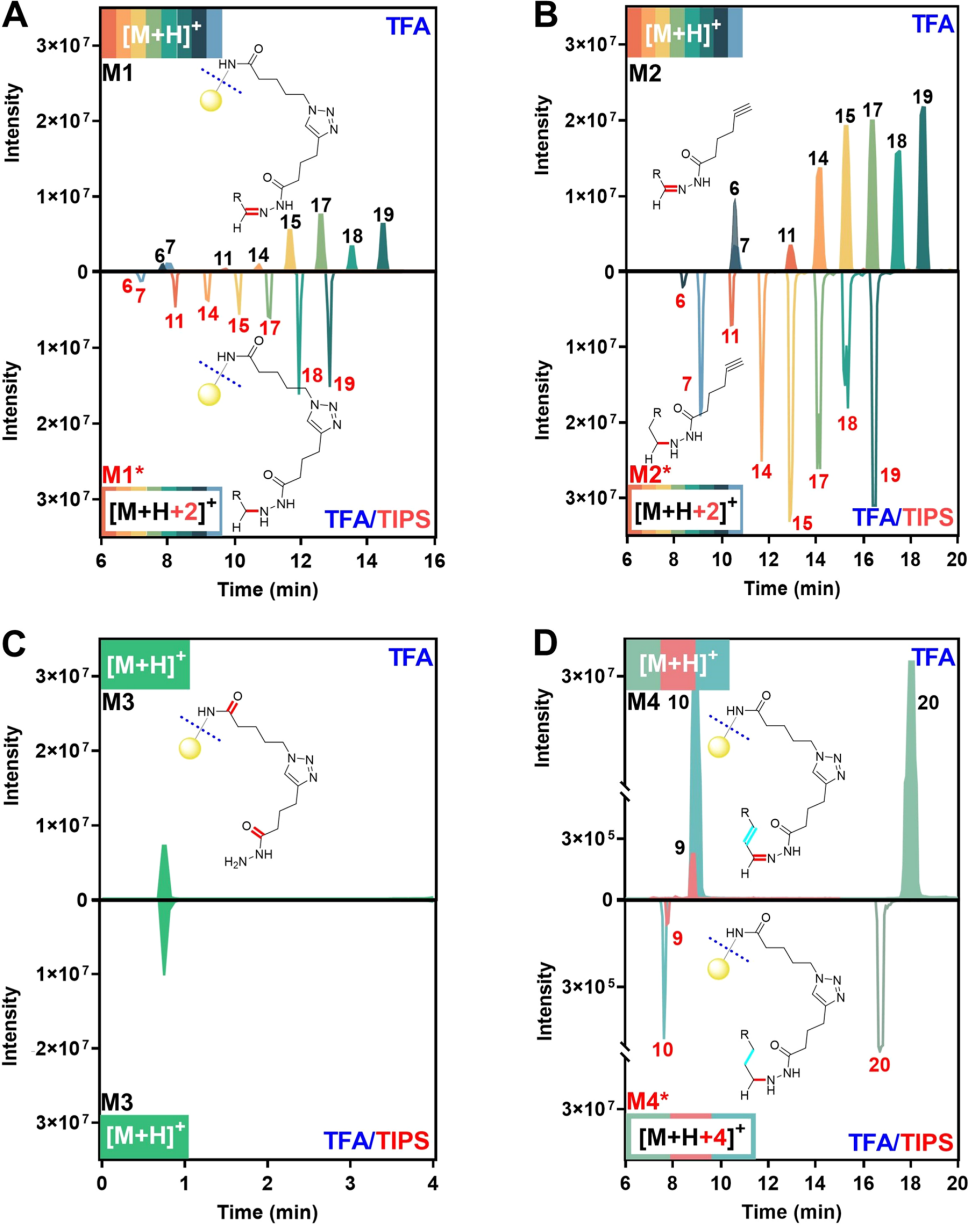
Influence of TIPS in cleavage solvents on the detection of carbonyl metabolites using GDBE method. (A) EICs of eight mixed carbonyl standards without C=C bond (Nos. 6, 7, 11, 14, 15, 17, 18, and 19) after GDBE treatment and cleavage by TFA ([M + H]^+^, above) and TFA/TIPS ([M + H + 2]^+^, below). (B) EICs of mixed carbonyl standards without C=C bond after the derivatization with alkyne hydrazide and treatment of TFA ([M + H]^+^, above) or TFA/TIPS ([M + H + 2]^+^, below). (C) EICs of the cleaved product from CCRI resins by TFA or TFA/TIPS. (D) EICs of three carbonyl standards with C=C bond (Nos. 9, 10, and 20) after GDBE treatment and cleavage by TFA ([M + H]^+^, above) and TFA/TIPS ([M + H + 4]^+^, below). The chemical structures of potential analytes detected are shown in Figures S10–S13.

Our immobilized CCRI resin offers a significant advantage with its ability to selectively cleave the C–N bond (indicated by the dashed line in Figure 1A), under acidic conditions, resulting in the formation of hydrazone derivatives for LC-MS analysis. The common reagent mixture of TFA/TIPS/water (*v*/*v*/*v*, 95:2.5:2.5) has been widely used for cleaving the NovaPEG Rink Amide resin.^15,21^ In this study, when the cleavage solution contained TFA/TIPS, the expected hydrazone derivatives derived from carbonyl metabolites without a C=C bond were not detected in the form of [M + H]^+^ ions. However, their [M + H + 2]^+^ ions were observed. Conversely, when only TFA was used as the cleavage solution, the desired hydrazone derivatives ([M + H]^+^ ions) were successfully detected (Figures 3A and S10). A similar phenomenon was also observed when carbonyl metabolites were derivatized with alkyne hydrazide and treated with TFA/TIPS (Figures 3B and S11). One plausible explanation for this observation is that TIPS, being a mild reductant, may lead to the reduction of hydrazone derivatives containing C=O and C=N bonds. To test this hypothesis and further elucidate the structure of the reduction products, the cleaved products of CCRI resin using either TFA or TFA/TIPS were analyzed, and identical cleaved products in the form of [M + H]^+^ ion were detected (Figures 3C and S12). This indicates that the possibility of a C=O reduction can be excluded. Thus, it is inferred that the reduction of hydrazone derivatives might occur at the C=N bond of the hydrazone moiety. To further investigate whether TIPS could reduce the C=C bond originating from carbonyl compounds, three carbonyl metabolites (Nos. 9, 10, and 20) with conjugated C=C bonds were tested. As expected, upon treatment with TFA/TIPS, their [M + H + 4]^+^ ions were observed, indicating that both the C=N and C=C bonds are reduced by TIPS (Figures 3D and S13). Taken together, the addition of TIPS in the cleavage solution is inappropriate for releasing the desired hydrazone derivatives with C=N and conjugated C=C bonds.

### High Selectivity and Enrichment Performance of the GDBE Method

**Figure 4:**
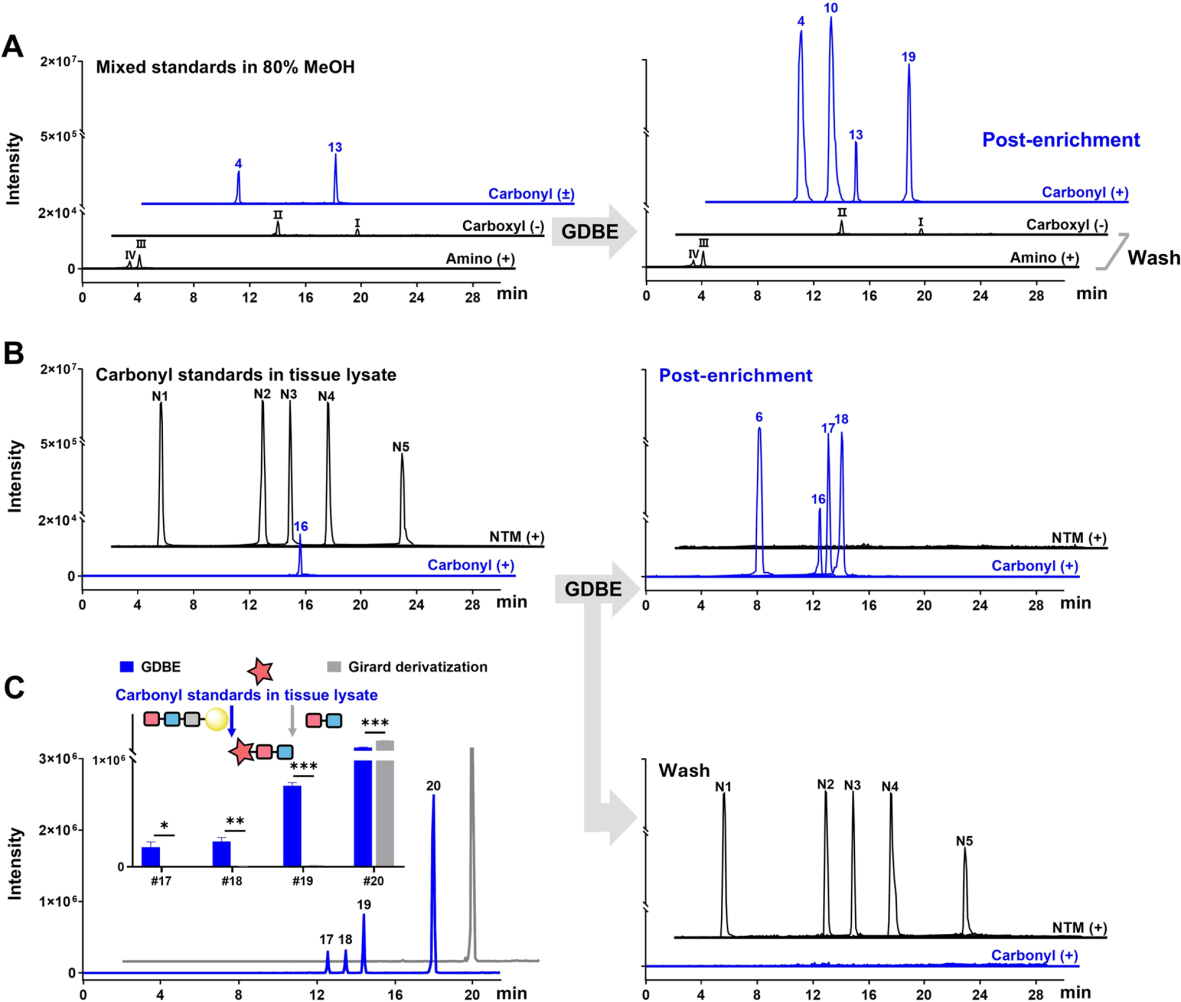
Chemoselectivity and enrichment performance of the GDBE strategy. LC-MS chromatograms for enrichment experiments with (A) the given mixed standards in 80% MeOH and (B) carbonyl standards in liver tissue lysate. Peaks of nontargeted standards without carbonyl group (I–IV) and nontargeted metabolites (NTM, N1–N5) detected in the pre-enrichment solution are absent following enrichment by GDBE treatment but remain in the washing eluent. The mixed standards are composed of carbonyl metabolites (Nos. 4, 10, 13, and 19), and noncarbonyl compounds, including carboxyl-containing compounds (I, dodecanoic acid; II, 5-phenylvaleric acid) and amine-containing compounds (III, phenylethylamine; IV, 5-hydroxytryptamine). Their chemical structures are shown in Figure S14. The carbonyl standards are composed of Nos. 6, 16, 17, and 18, and the nontargeted metabolites (NTM) include N1–N5: N1, *t*_R_ 3.52 min_*m*/*z* 120.0806; N2, *t*_R_ 10.49 min_*m*/*z* 462.2647; N3, *t*_R_ 12.42 min_*m*/*z* 355.2610; N4, *t*_R_ 14.99 min_*m*/*z* 478.2873; N5, *t*_R_ 20.13 min_*m*/*z* 263.2358. (C) The influence of GDBE on matrix effects. Mixed carbonyl standards were added to liver tissue lysate and subjected to CCRI resin (GDBE) or resin acidic hydrolysis product (Girard derivatization). Detailed information on carbonyl standards is listed in Table 1.

To assess the selectivity and enrichment performance of the GDBE method, a standard mixture and liver tissue lysate were used. First, a standard mixture was prepared by adding endogenous metabolite standards without carbonyl group (Figure S14), including two carboxylic acids (dodecanoic acid and 5-phenylvaleric acid, detected in negative ion mode) and two amines (phenylethylamine and 5-hydroxytryptamine, detected in positive ion mode), to a solution containing four carbonyl standards (Nos. 4, 10, 13, and 19). After GDBE treatment, the post-enrichment solution and the washing eluent were collected and analyzed by LC-MS. As shown in Figure 4A, the four small molecules without a carbonyl group (I–V) were detected in pre-enrichment solution but were absent following GDBE enrichment, remaining in the washing eluent. In contrast, the carbonyl standards in hydrazone-derived form were exclusively detected in the post-enrichment solutions. The hydrazide group is capable of conjugating to carboxylic acids, but this process requires the use of 1-chloro-4-methylpyridinium iodide (CMPI) as an activator under slightly alkaline conditions.^24^ Given the absence of these necessary components under the present conditions, the possibility of a reaction occurring between the CCRI resin and carboxylic metabolites can be excluded. Similar results were observed in liver tissue lysate spiked with other carbonyl standards (Figure 4B). After GDBE, several representative nontargeted metabolites (N1–N5) detected in liver tissue lysate were absent in the post-enrichment solution but remained in the washing eluent. In contrast, the carbonyl metabolites in their hydrazone-derived form were clearly visible after GDBE. Collectively, these results indicate that the developed GDBE method is highly selective for capturing carbonyl metabolites in biological samples.

The CCRI resin allows for the selective capture of carbonyl metabolites, facilitating their isolation from the complex sample matrix. This is predicted to simplify the mass spectra by reducing the matrix interference. To investigate the influence of GDBE on matrix effects, four representative carbonyl standards (1 μM each) were added to liver tissue lysate and subjected to either CCRI resin labeling (GDBE) or resin acidic hydrolysis product labeling (Girard derivatization). Except for hexadecenal (#20), the signal responses of the carbonyl standards following GDBE were significantly higher compared to those obtained from Girard derivatization alone (Figure 4C). These results indicated that the chemoselective strategy enables the removal of unwanted metabolites and facilitates the capture and enrichment of metabolites of interest from the highly complicated matrix, thereby increasing resistance to matrix effects. This finding also suggests that combination derivatization with selective enrichment is essential for the detection of carbonyls in biological samples by LC-MS.

Additionally, the same carbonyl standards were added to either an 80% methanol solution or liver tissue lysate and subjected to CCRI resin labeling. The signal responses of most carbonyl standards in 80% methanol were comparable to those in tissue lysate, also indicating minimal matrix influence of GDBE (Figure S15).

### Profiling of Carbonyl Submetabolome in Liver Tissue from Alcohol-Fed Mice

**Figure 5:**
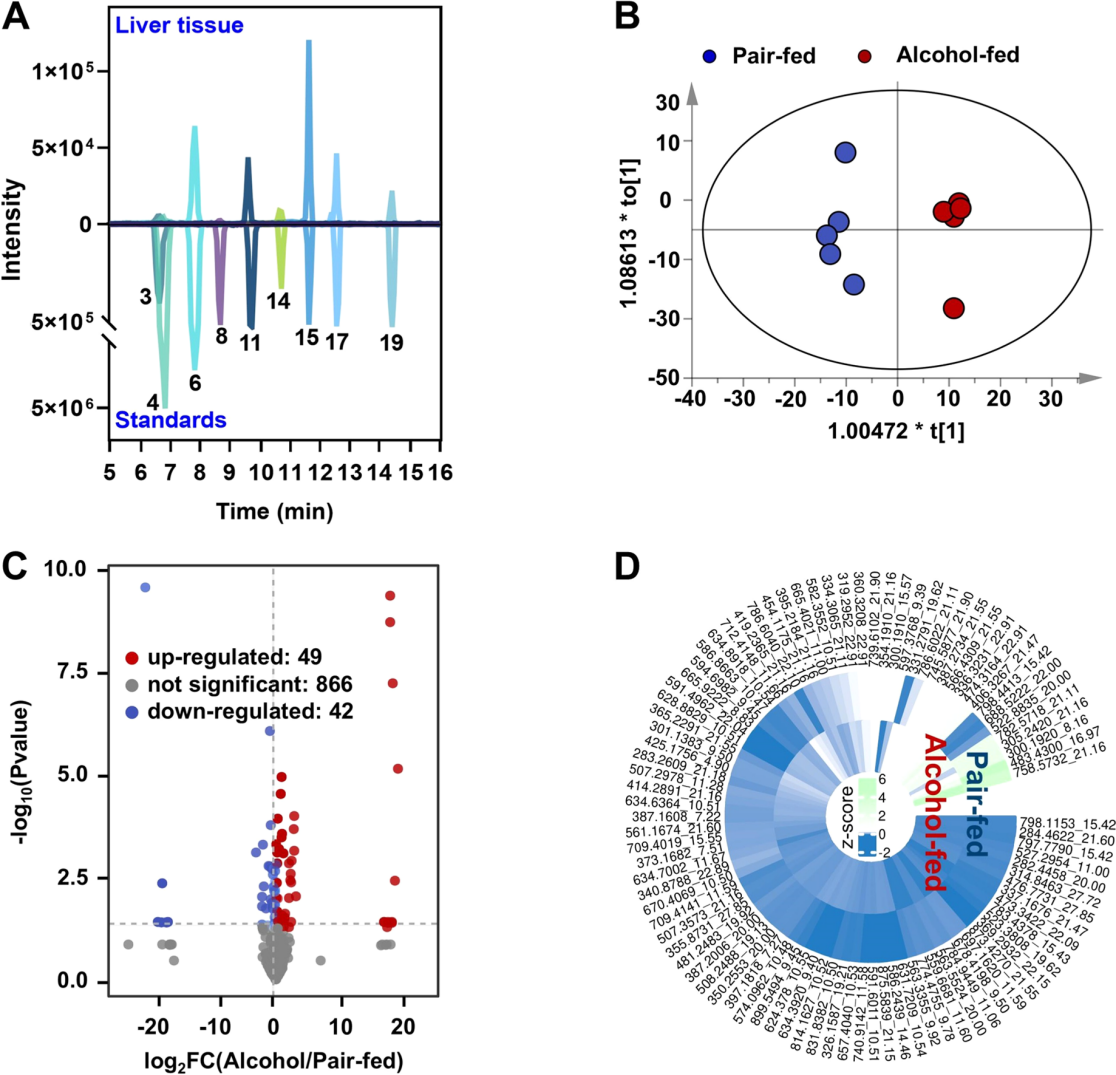
Application of the GDBE strategy for nontargeted profiling of the carbonyl submetabolome in liver tissue from alcohol-fed mice. (A) Nine carbonyl metabolites were unambiguously confirmed using their commercial standards. The numbers of carbonyl standards are represented in the same manner as in Table 1. (B) OPLS-DA score plot based on carbonyl metabolites in liver tissues from pair-fed mice and alcohol-fed mice. (C) Volcano plot indicating up-regulated (red) and down-regulated (blue) carbonyl metabolites. (D) Z-score plot measuring the relative content of differential metabolites between groups.

The optimized GDBE was utilized to profile the carbonyl submetabolome in liver tissue from alcohol-fed and pair-fed mice. The liver tissue was processed by grinding it in 80% MeOH, and the resulting supernatant was then subjected to CCRI resin for enrichment. After thorough washing, carbonyl-bound resin was cleaved using TFA, and the released hydrazone derivatives were subsequently analyzed using LC-MS with MS^E^ mode (Figure 1C), which allows for the accurate measurement of precursor and product ions through alternating low- and high-energy collision-induced dissociation.^27,28^ The detailed workflow of the discovery and annotation of carbonyl metabolites is illustrated in Figure S16. The raw data, acquired in positive ion mode, were directly processed by UNIFI software. To eliminate potential signals originating from the resin, we set a mass signal threshold of 5000 for metabolites, and specifically selected ions featuring a reporter ion fragment (*m*/*z* 100.0757, [C_5_H_10_NO]) in the MS/MS spectra using the UNIFI discovery module. The presence of an additional characteristic fragment with a *m*/*z* of 237.1352 further facilitates the recognition of carbonyl metabolites. These screened ions were further filtered with a *m*/*z* value greater than 281.1721, which corresponds to the signal of methanal-derived hydrazone derivatives, the minimum *m*/*z* of carbonyl derivatives. Following this workflow, a total of 957 ions were highlighted as potential carbonyl metabolites in liver tissue from the mice. By subtracting the mass of hydrazone derivatives (*m*/*z* 251.1615, [C_11_H_19_N_6_O]), we obtained the accurate masses of the corresponding native carbonyl metabolites. These metabolites were annotated using their accurate mass and structure-related fragments by matching them with biochemical databases, including MycompoundID (http://www.mycompoundid.org) and Human Metabolome Database (https://hmdb.ca/), with a MS tolerance of 20 ppm.^21,26^ As a result, 101 ions were searchable in the HMDB. After exogenous metabolites and duplicates were excluded, 76 carbonyl metabolites were temporarily annotated (Table S1). However, many highlighted ions remained unsearchable, likely due to incomplete data and novel carbonyl compounds present in biological samples. Metabolite identification continues to pose significant challenges in both untargeted metabolomics and chemical derivatization-assisted submetabolomics, which may be potentially resolved by artificial intelligence (AI)-assisted approaches. Notably, 9 carbonyl compounds were unambiguously confirmed using their commercial standards through comparing the retention times and fragment ions (Figure 5A), demonstrating that the GDBE method is reliable for profile carbonyl metabolites in complicated biological samples.

Carbonyl submetabolome was further compared between liver tissues from alcohol-fed and pair-fed mice using the GDBE method. The application of OPLS-DA analysis revealed a distinct separation between the two groups based on their carbonyl profiles in the liver (R^2^X = 0.378, R^2^Y = 0.99, Q^2^ = 0.623) (Figure 5B), all the samples are in the 95% confidence interval (Hotelling’s T-squared Ellipse). A total of 91 carbonyl metabolites (FC > 2, VIP > 1 and *p* < 0.05) were highlighted by the volcano plot (Figure 5C), and their level differences were displayed in Z-score plot (Figure 5D). Long-term alcohol exposure leads to an excessive production of reactive oxygen species (ROS) and other prooxidants, resulting in oxidative stress in various organs, particularly in the liver where ethanol is primarily metabolized to acetaldehyde.^17,29,30^ The ROS generated by alcohol consumption can be highly detrimental to hepatocytes due to their ability to induce oxidative modifications in cellular macromolecules, including DNA damage, protein denaturation, and lipid peroxidation of cellular membranes.^30^ Many carbonyl metabolites are considered secondary oxidation products that arise from lipid peroxidation following oxidative stress.^2^ Hence, the observed increase in carbonyl metabolites in alcohol-fed mice may be attributed to the elevated levels of ROS induced by alcohol consumption.

## Conclusions

In summary, our study presents a Girard derivatization-based enrichment strategy for comprehensive profiling of carbonyl metabolites by LC-MS. The GDBE strategy demonstrates high selectivity in capturing and enriching carbonyl metabolites from complex matrices and can significantly enhance the MS sensitivity for target analytes. Additionally, the GDBE strategy facilitates structural recognition of carbonyl derivatives by a high diagnostic reporter-ion with a *m*/*z* 100.0757 in positive ion mode. Through the implementation of the GDBE method, we successfully identified a total of 957 potential carbonyl metabolites in liver tissue from mice. Among them, 76 carbonyl metabolites were annotated, indicating the potential of this strategy for the efficient nontargeted profiling of the carbonyl submetabolome in complex biological samples. It should be noted that such a strategy may not be suitable for unstable carbonyl metabolites under acidic conditions. This strategy provides a valuable tool in advancing our understanding of carbonyl metabolites and their implications in various biological processes.

## Supporting Information Available

The chemical structures of 20 carbonyl standards, the synthesis of CCRI resins, optimization of Cu(I)-catalyzedazide–alkyne cycloaddition (CuAAC) reaction conditions for the synthesis of CCRI resin, the regeneration of the immobilized CCRI resin, the synthesis of carboxyl or thiol capture and reporter-ion installation resins, optimization of Girard derivatization conditions, cleavage reaction conditions and redissolve solvent using 6 representative carbonyl standards, optimization of LC-MS conditions using 6 representative carbonyl standards, effects of drying step during sample processing on the signal of carbonyl metabolites using GDBE method, MS/MS spectra and fragmentation patterns of carbonyl metabolites in the form of hydrazone derivative detected in the positive ion mode, the chemical structures of the hydrazone derivatives derived from carbonyl metabolites without a C=C bond (M1) and their possible reduction products (M1*), the chemical structures of alkyne hydrazide-derivatized carbonyls (M2) and their possible reduction products (M2*), the chemical structures of the cleaved products of CCRI resin, the chemical structures of the expected hydrazone derivatives derived from carbonyl metabolites with a C=C bond (M4) and their possible reduction products (M4*), chemical structures of endogenous metabolite standards without carbonyl group, the influence of GDBE on matrix effects, the workflow of comprehensive profiling and relative quantification for the discovery and annotation of carbonyl metabolites from mice liver using GDBE strategy (PDF)
