# Supplementary material for: Girard Derivatization-Based Enrichment Strategy for Profiling the Carbonyl Submetabolome in Biological Samples
Source: Anal Chem. 2024 Dec 19;96(52):20414–24. doi: 10.1021/acs.analchem.4c04037 (PMC11696826; doi:10.1021/acs.analchem.4c04037)
Supplement: Supplementary file 1 — ac4c04037_si_001.pdf [file ac4c04037_si_001.pdf]

Supporting Information

Girard Derivatization-Based Enrichment Strategy for Profiling the Carbonyl Submetabolome in Biological Samples

Xin Tao [a], Jia-Yue Liu [a], Jun-Yi Zhou [a], Jiang-Kun Dai [a], Ze-Yu Xiao [b], Hou-Kai Li [c], Jian-Bo Wan [a,*]

[a] State Key Laboratory of Quality Research in Chinese Medicine, Institute of Chinese Medical Sciences, University of Macau, Taipa, Macao SAR, China

[b] Collaborative Translational Medicine Collaborative Innovation Center, Department of Pharmacology and Chemical Biology, Institute of Medical Sciences, Shanghai Jiao Tong University School of Medicine, Shanghai, China

[c] School of Pharmacy, Shanghai University of Traditional Chinese Medicine, Shanghai, China.

***Correspondence:**

Prof. Jian-Bo Wan,

E-mail: jbwan@um.edu.mo

Room 6034, Building N22, Institute of Chinese Medical Sciences,

University of Macau, Avenida da Universidade,

Taipa, Macao SAR, China

Table of Content

Table S1. Potential carbonyl compounds detected in the liver tissue of mice....................................S3

Figure S1. The chemical structures of 20 carbonyl standards...S8

Figure S2. The synthesis of CCRI resins using a two-step process..S9

Figure S3. Optimization of Cu(I)-catalyzedazide-alkyne cycloaddition (CuAAC) reaction conditions for the synthesis of CCRI resin..S10

Figure S4. The regeneration of the immobilized CCRI resin..S11

Figure S5. The synthesis of carboxyl or thiol capture and reporter-ion installation resins........S12

Figure S6. Optimization of Girard derivatization conditions, cleavage reaction conditions and redissolve solvent using 6 representative carbonyl standards...S13

Figure S7. Optimization of LC-MS conditions using 6 representative carbonyl standards.............S14

Figure S8. Effects of drying step during sample processing on the signal of carbonyl metabolites using GDBE method...S15

Figure S9. MS/MS spectra and fragmentation patterns of carbonyl metabolites in the form of hydrazone derivative detected in the positive ion mode...S16

Figure S10. The chemical structures of the hydrazone derivatives derived from carbonyl metabolites without a C=C bond (M1) and their possible reduction products (M1*)...S17

Figure S11. The chemical structures of alkyne hydrazide-derivatized carbonyls (M2) and their possible reduction products (M2*)...S18

Figure S12. The chemical structures of the cleaved products of CCRI resin.................................S19

Figure S13. The chemical structures of the expected hydrazone derivatives derived from carbonyl metabolites with a conjugated C=C bond (M4) and their possible reduction products (M4*)..........S20

Figure S14. Chemical structures of endogenous metabolite standards without carbonyl group, including carboxylic acids (I and II) and amines (III and IV) in the selectivity experiments...........S21

Figure S15. The influence of GDBE on matrix effects...S22

Figure S16. The workflow of comprehensive profiling and relative quantification for the discovery and annotation of carbonyl metabolites from mice liver using GDBE strategy...............................S23

Table S1. Potential carbonyl compounds detected in the liver tissue of mice

No.	RT (min)	Observed *m/z*	Accurate mass	HMDB No.	Candidate	Formula	Adduct ion	MS Error (ppm)	Common Fragment Ions Found
1	4.57	425.1762	192.0270	HMDB05971	Diketogulonic acid	$C_6H_8O_7$	$[M+H-H_2O]^+$	-4	100.07569 $[C_5H_{10}NO]$
			174.0164	HMDB01264	Dehydroascorbic acid	$C_6H_6O_6$	$[M+H]^+$	-4	100.07569 $[C_5H_{10}NO]$
2	4.62	477.2546	208.0848	HMDB12948	Formyl-5-hydroxykynurenamine	$C_{10}H_{12}N_2O_3$	$[M+H+H_2O]^+$	-5	100.07569 $[C_5H_{10}NO]$
3	4.63	424.2275	173.0688	HMDB06488	N-Acetyl-L-glutamate 5-semialdehyde	$C_7H_{11}NO_4$	$[M+H]^+$	-7	100.07569 $[C_5H_{10}NO]$
4	4.64	325.1590	74.0004	HMDB00119	Glyoxylic acid	$C_2H_2O_3$	$[M+H]^+$	-9	100.07569 $[C_5H_{10}NO]$
5	4.99	364.2064	131.0582	HMDB02104	L-Glutamic-gamma-semialdehyde	$C_5H_9NO_3$	$[M+H-H_2O]^+$	-7	100.07569 $[C_5H_{10}NO]$
6	4.99	396.1955	145.0375	HMDB01552	2-Keto-glutaramic acid	$C_5H_7NO_4$	$[M+H]^+$	-9	100.07569 $[C_5H_{10}NO]$
7	5.35	365.1513	132.0059	HMDB00223	Oxalacetic acid	$C_4H_4O_5$	$[M+H-H_2O]^+$	-14	100.07569 $[C_5H_{10}NO]$
8	5.36	364.2070	131.0582	HMDB01149	5-Aminolevulinic acid	$C_5H_9NO_3$	$[M+H-H_2O]$	-6	100.07569 $[C_5H_{10}NO]$
			131.0582	HMDB06272	5-Amino-2-oxopentanoic acid	$C_5H_9NO_3$	$[M+H-H_2O]^+$	-6	100.07569 $[C_5H_{10}NO]$
9	5.36	425.1757	192.0270	HMDB06511	2,3-Diketo-L-gulonate	$C_6H_8O_7$	$[M+H-H_2O]^+$	-5	100.07569 $[C_5H_{10}NO]$
10	5.42	447.2544	178.0742	HMDB01267	4-Oxo-4-(3-pyridyl)-butanamide	$C_9H_{10}N_2O_2$	$[M+H+H_2O]^+$	19	100.07569 $[C_5H_{10}NO]$
11	5.65	339.1737	88.0160	HMDB00243	Pyruvic acid	$C_3H_4O_3$	$[M+H]^+$	-11	100.07569 $[C_5H_{10}NO]$
			167.9729	HMDB04045	3-Sulfopyruvic acid	$C_3H_4O_6S$	$[M+H-SO_3]^+$	-9	100.07569 $[C_5H_{10}NO]$
			88.0160	HMDB11111	Malonic semialdehyde	$C_3H_4O_3$	$[M+H]^+$	-11	100.07569 $[C_5H_{10}NO]$
12	5.66	425.1756	192.0270	HMDB05971	Diketogulonic acid	$C_6H_8O_7$	$[M+H-H_2O]^+$	-5	100.07569 $[C_5H_{10}NO]$
13	6.05	426.2441	158.0579	HMDB00635	Succinylacetone	$C_7H_{10}O_4$	$[M+NH_4]^+$	-5	100.07569 $[C_5H_{10}NO]$
14	6.12	459.2435	208.0848	HMDB00684	L-Kynurenine	$C_{10}H_{12}N_2O_3$	$[M+H]^+$	-6	100.07569 $[C_5H_{10}NO]$
			208.0848	HMDB12948	Formyl-5-hydroxykynurenamine	$C_{10}H_{12}N_2O_3$	$[M+H]^+$	-6	100.07569 $[C_5H_{10}NO]$
			252.0746	HMDB04086	5-Hydroxy-N-formylkynurenine	$C_{11}H_{12}N_2O_5$	$[M+H-CO_2]^+$	-5	100.07569 $[C_5H_{10}NO]$
15	6.43	503.3187	270.1620	HMDB00145	Estrone	$C_{18}H_{22}O_2$	$[M+H-H_2O]^+$	11	100.07569 $[C_5H_{10}NO]$
16	6.45	371.1567	119.9881	HMDB01368	3-Mercaptopyruvic acid	$C_3H_4O_3S$	$[M+H]^+$	19	100.07569 $[C_5H_{10}NO]$
17*	6.73	403.2055	152.0473	HMDB12308	Vanillin	$C_8H_8O_3$	$[M+H]^+$	-8	100.07569 $[C_5H_{10}NO]$
18*	6.78	401.1926	150.0317	HMDB0246430	4-Formylbenzoic acid	$C_8H_6O_3$	$[M+H]^+$	-1	100.07569 $[C_5H_{10}NO]$
19	6.85	321.1984	88.0524	HMDB03243	Acetoin	$C_4H_8O_2$	$[M+H-H_2O]^+$	-15	100.07569 $[C_5H_{10}NO]$

20	7.21	426.2306	175.0633	HMDB04073	5-Hydroxyindoleacetaldehyde	$C_{10}H_9NO_2$	$[M+H]^+$	13	100.07569 [$C_5H_{10}NO$]
21	7.21	397.1805	146.0215	HMDB00208	Oxoglutaric acid	$C_5H_6O_5$	$[M+H]^+$	-6	100.07569 [$C_5H_{10}NO$]
			190.0114	HMDB03974	Oxalosuccinic acid	$C_6H_6O_7$	$[M+H-CO_2]$	-6	100.07569 [$C_5H_{10}NO$]
22	7.47	518.3166	250.1205	HMDB02012	Ubiquinone Q1	$C_{14}H_{18}O_4$	$[M+NH_4]$	16	100.07569 [$C_5H_{10}NO$]
23	7.58	555.2296	286.0477	HMDB05800	Luteolin	$C_{15}H_{10}O_6$	$[M+H+H_2O]^+$	18	100.07569 [$C_5H_{10}NO$]
			286.0477	HMDB05801	Kaempferol	$C_{15}H_{10}O_6$	$[M+H+H_2O]^+$	18	100.07569 [$C_5H_{10}NO$]
24*	7.84	357.2007	106.0419	HMDB06115	Benzaldehyde	C_7H_6O	$[M+H]^+$	-8	100.07569 [$C_5H_{10}NO$]
25	8.17	415.2314	326.1213	HMDB06590	2-O-a-L-Fucopyranosyl-galactose	$C_{12}H_{22}O_{10}$	$[M+H-C_6H_{10}O_5]^+$	2	100.07569 [$C_5H_{10}NO$]
			326.1213	HMDB06701	3-O-a-L-Fucopyranosyl-D-glucose	$C_{12}H_{22}O_{10}$	$[M+H-C_6H_{10}O_5]^+$	2	100.07569 [$C_5H_{10}NO$]
			146.0579	HMDB06900	(S)-2-Aceto-2-hydroxybutanoic acid	$C_6H_{10}O_4$	$[M+H+H_2O]^+$	4	100.07569 [$C_5H_{10}NO$]
			164.0685	HMDB10207	L-Rhamnulose	$C_6H_{12}O_5$	$[M+H]^+$	3	100.07569 [$C_5H_{10}NO$]
26*	8.71	351.2461	100.0888	HMDB05994	Hexanal	$C_6H_{12}O$	$[M+H]^+$	-12	100.07569 [$C_5H_{10}NO$]
27	8.71	371.2165	164.0473	HMDB00205	Phenylpyruvic acid	$C_9H_8O_3$	$[M+H-CO_2]^+$	-7	100.07569 [$C_5H_{10}NO$]
28	9.35	603.3441	370.1814	HMDB02759	Androsterone sulfate	$C_{19}H_{30}O_5S$	$[M+H-H_2O]$	19	100.07569 [$C_5H_{10}NO$]
			334.1700	HMDB04634	4-Chloromethandienone	$C_{20}H_{27}ClO_2$	$[M+H+H_2O]^+$	4	100.07569 [$C_5H_{10}NO$]
			370.1814	HMDB06278	5a-Dihydrotestosterone sulfate	$C_{19}H_{30}O_5S$	$[M+H-H_2O]^+$	19	100.07569 [$C_5H_{10}NO$]
29	9.39	597.3768	364.2250	HMDB06754	11b,21-Dihydroxy-3,20-oxo-5b-pregnan-18-al	$C_{21}H_{32}O_5$	$[M+H-H_2O]^+$	1	100.07569 [$C_5H_{10}NO$]
			364.2250	HMDB06760	11b,17a,21-Trihydroxypreg-nenolone	$C_{21}H_{32}O_5$	$[M+H-H_2O]^+$	1	100.07569 [$C_5H_{10}NO$]
			364.2250	HMDB12460	11b,17a,21-Trihydroxy-5b-pregnane-3,20-dione	$C_{21}H_{32}O_5$	$[M+H-H_2O]^+$	1	100.07569 [$C_5H_{10}NO$]
			346.2144	HMDB00015	Cortexolone	$C_{21}H_{30}O_4$	$[M+H]^+$	1	100.07569 [$C_5H_{10}NO$]
			346.2144	HMDB01547	Corticosterone	$C_{21}H_{30}O_4$	$[M+H]^+$	1	100.07569 [$C_5H_{10}NO$]
			346.2144	HMDB04030	21-Deoxycortisol	$C_{21}H_{30}O_4$	$[M+H]^+$	1	100.07569 [$C_5H_{10}NO$]
			346.2144	HMDB06756	21-Hydroxy-5b-pregnane-3,11,20-trione	$C_{21}H_{30}O_4$	$[M+H]^+$	1	100.07569 [$C_5H_{10}NO$]
30	9.41	625.3726	374.2093	HMDB11651	11beta,20-Dihydroxy-3-oxopregn-4-en-21-oic acid	$C_{22}H_{30}O_5$	$[M+H]^+$	3	100.07569 [$C_5H_{10}NO$]
31	9.43	385.2316	117.0426	HMDB06454	L-2-Amino-3-oxobutanoic acid	$C_4H_7NO_3$	$[M+NH4]^+$	3	100.07569 [$C_5H_{10}NO$]
			117.0426	HMDB12249	L-Aspartate-semialdehyde	$C_4H_7NO_3$	$[M+NH4]^+$	3	100.07569 [$C_5H_{10}NO$]
32	9.44	391.2393	158.0943	HMDB10721	3-Oxooctanoic acid	$C_8H_{14}O_3$	$[M+H-H_2O]^+$	-15	100.07569 [$C_5H_{10}NO$]
33	9.45	611.3834	342.2042	HMDB02277	2,3-Dinor-6-keto-prostaglandin F1 a	$C_{18}H_{30}O_6$	$[M+H+H_2O]^+$	12	100.07569 [$C_5H_{10}NO$]
34	9.47	613.3717	362.2093	HMDB00063	Cortisol	$C_{21}H_{30}O_5$	$[M+H]^+$	1	100.07569 [$C_5H_{10}NO$]

			362.2093	HMDB00319	18-Hydroxycorticosterone	$C_{21}H_{30}O_5$	$[M+H]^+$	1	100.07569 [$C_5H_{10}NO$]
			344.1988	HMDB04029	11-Dehydrocorticosterone	$C_{21}H_{28}O_4$	$[M+H+H_2O]^+$	1	100.07569 [$C_5H_{10}NO$]
			362.2093	HMDB06758	17a,21-Dihydroxy-5b-pregnane-3,11,20-trione	$C_{21}H_{30}O_5$	$[M+H]^+$	1	100.07569 [$C_5H_{10}NO$]
35*	9.64	365.2625	114.1045	HMDB31475	Heptanal	$C_7H_{14}O$	$[M+H]^+$	-10	100.07569 [$C_5H_{10}NO$]
36	9.78	586.3621	318.1831	HMDB06709	Ubiquinone Q2	$C_{19}H_{26}O_4$	$[M+NH_4]^+$	-16	100.07569 [$C_5H_{10}NO$]
37	9.81	453.1744	220.0249	HMDB12236	Imidazole acetol-phosphate	$C_6H_9N_2O_5P$	$[M+H-H_2O]^+$	-3	100.07569 [$C_5H_{10}NO$]
38	9.81	569.3469	300.1725	HMDB00010	2-Methoxyestrone	$C_{19}H_{24}O_3$	$[M+H+H_2O]^+$	4	100.07569 [$C_5H_{10}NO$]
			318.1831	HMDB06709	Ubiquinone Q2	$C_{19}H_{26}O_4$	$[M+H]^+$	4	100.07569 [$C_5H_{10}NO$]
39	9.82	601.3772	350.2093	HMDB02341	8-iso-15-keto-PGE2	$C_{20}H_{30}O_5$	$[M+H]^+$	11	100.07569 [$C_5H_{10}NO$]
			350.2093	HMDB02664	Prostaglandin E3	$C_{20}H_{30}O_5$	$[M+H]^+$	11	100.07569 [$C_5H_{10}NO$]
			350.2093	HMDB03034	Prostaglandin D3	$C_{20}H_{30}O_5$	$[M+H]^+$	11	100.07569 [$C_5H_{10}NO$]
			350.2093	HMDB03175	15-Keto-prostaglandin E2	$C_{20}H_{30}O_5$	$[M+H]^+$	11	100.07569 [$C_5H_{10}NO$]
40	9.87	547.3350	314.1882	HMDB06285	4-oxo-Retinoic acid	$C_{20}H_{26}O_3$	$[M+H-H_2O]^+$	-7	100.07569 [$C_5H_{10}NO$]
41	9.87	577.3519	344.1988	HMDB04029	11-Dehydrocorticosterone	$C_{21}H_{28}O_4$	$[M+H-H_2O]^+$	4	100.07569 [$C_5H_{10}NO$]
42	9.93	595.3672	362.2093	HMDB06758	17a,21-Dihydroxy-5b-pregnane-3,11,20-trione	$C_{21}H_{30}O_5$	$[M+H-H_2O]^+$	11	100.07569 [$C_5H_{10}NO$]
			344.1988	HMDB04029	11-Dehydrocorticosterone	$C_{21}H_{28}O_4$	$[M+H]^+$	12	100.07569 [$C_5H_{10}NO$]
43	9.94	541.3379	308.1776	HMDB02720	Gestrinone	$C_{21}H_{24}O_2$	$[M+H-H_2O]^+$	17	100.07569 [$C_5H_{10}NO$]
44	9.94	441.2677	173.0800	HMDB04225	2-Oxoarginine	$C_6H_{11}N_3O_3$	$[M+NH_4]^+$	-1	100.07569 [$C_5H_{10}NO$]
45	10.48	591.3631	340.2038	HMDB03033	Canrenone	$C_{22}H_{28}O_3$	$[M+H]^+$	-4	100.07569 [$C_5H_{10}NO$]
46	10.48	750.4487	482.2516	HMDB10351	11-beta-hydroxyandrosterone-3-glucuronide	$C_{25}H_{38}O_9$	$[M+NH_4]^+$	12	100.07569 [$C_5H_{10}NO$]
47	10.50	627.3870	376.2250	HMDB11652	11beta-Hydroxy-3,20-dioxopregn-4-en-21-oic acid	$C_{22}H_{32}O_5$	$[M+H]^+$	1	100.07569 [$C_5H_{10}NO$]
48	10.51	619.3747	368.2199	HMDB01908	19-Hydroxy-PGE2	$C_{20}H_{32}O_6$	$[M+H]^+$	-11	100.07569 [$C_5H_{10}NO$]
			368.2199	HMDB01979	6,15-Diketo,13,14-dihydro-PGF1a	$C_{20}H_{32}O_6$	$[M+H]^+$	-11	100.07569 [$C_5H_{10}NO$]
			350.2093	HMDB02341	8-iso-15-keto-PGE2	$C_{20}H_{30}O_5$	$[M+H+H_2O]^+$	-11	100.07569 [$C_5H_{10}NO$]
			350.2093	HMDB02664	Prostaglandin E3	$C_{20}H_{30}O_5$	$[M+H+H_2O]^+$	-11	100.07569 [$C_5H_{10}NO$]
			350.2093	HMDB03175	15-Keto-prostaglandin E2	$C_{20}H_{30}O_5$	$[M+H+H_2O]^+$	-11	100.07569 [$C_5H_{10}NO$]
			368.2199	HMDB03247	20-Hydroxy-PGE2	$C_{20}H_{32}O_6$	$[M+H]^+$	-11	100.07569 [$C_5H_{10}NO$]
			368.2199	HMDB04241	6-Ketoprostaglandin E1	$C_{20}H_{32}O_6$	$[M+H]^+$	-11	100.07569 [$C_5H_{10}NO$]
			368.2199	HMDB12110	5(6)-Epoxy Prostaglandin E1	$C_{20}H_{32}O_6$	$[M+H]^+$	-11	100.07569 [$C_5H_{10}NO$]

49	10.52	421.2301	152.0586	HMDB04194	N1-Methyl-4-pyridone-3-carboxamide	$C_7H_8N_2O_2$	$[M+H+H_2O]^+$	-1	100.07569 [$C_5H_{10}NO$]
50	10.52	615.3785	364.2250	HMDB06754	11b,21-Dihydroxy-3,20-oxo-5b-pregnan-18-al	$C_{21}H_{32}O_5$	$[M+H]^+$	-13	100.07569 [$C_5H_{10}NO$]
			364.2250	HMDB00903	Tetrahydrocortisone	$C_{21}H_{32}O_5$	$[M+H]^+$	-13	100.07569 [$C_5H_{10}NO$]
			364.2250	HMDB03259	Dihydrocortisol	$C_{21}H_{32}O_5$	$[M+H]^+$	-13	100.07569 [$C_5H_{10}NO$]
			364.2250	HMDB06753	3a,11b,21-Trihydroxy-20-oxo-5b-pregnan-18-al	$C_{21}H_{32}O_5$	$[M+H]^+$	-13	100.07569 [$C_5H_{10}NO$]
			364.2250	HMDB06760	11b,17a,21-Trihydroxypreg-nenolone	$C_{21}H_{32}O_5$	$[M+H]^+$	-13	100.07569 [$C_5H_{10}NO$]
			364.2250	HMDB12460	11b,17a,21-Trihydroxy-5b-pregnane-3,20-dione	$C_{21}H_{32}O_5$	$[M+H]^+$	-13	100.07569 [$C_5H_{10}NO$]
			346.2144	HMDB04030	21-Deoxycortisol	$C_{21}H_{30}O_4$	$[M+H+H_2O]^+$	-13	100.07569 [$C_5H_{10}NO$]
			346.2144	HMDB06756	21-Hydroxy-5b-pregnane-3,11,20-trione	$C_{21}H_{30}O_4$	$[M+H+H_2O]^+$	-13	100.07569 [$C_5H_{10}NO$]
51	10.56	537.3079	286.1569	HMDB00313	16b-Hydroxyestrone	$C_{18}H_{22}O_3$	$[M+H]^+$	-19	100.07569 [$C_5H_{10}NO$]
			286.1569	HMDB00335	16a-Hydroxyestrone	$C_{18}H_{22}O_3$	$[M+H]^+$	-19	100.07569 [$C_5H_{10}NO$]
			286.1569	HMDB00343	2-Hydroxyestrone	$C_{18}H_{22}O_3$	$[M+H]^+$	-19	100.07569 [$C_5H_{10}NO$]
			286.1569	HMDB00406	16-Ketoestradiol	$C_{18}H_{22}O_3$	$[M+H]^+$	-19	100.07569 [$C_5H_{10}NO$]
			286.1569	HMDB05895	4-Hydroxyestrone	$C_{18}H_{22}O_3$	$[M+H]^+$	-19	100.07569 [$C_5H_{10}NO$]
52	10.58	671.4140	464.2410	HMDB03193	Testosterone glucuronide	$C_{25}H_{36}O_8$	$[M+H-CO_2]^+$	2	100.07569 [$C_5H_{10}NO$]
			464.2410	HMDB10327	Dehydroisoandrosterone 3-glucuronide	$C_{25}H_{36}O_8$	$[M+H-CO_2]^+$	2	100.07569 [$C_5H_{10}NO$]
			464.2410	HMDB10348	Dehydroepiandrosterone 3-glucuronide	$C_{25}H_{36}O_8$	$[M+H-CO_2]^+$	2	100.07569 [$C_5H_{10}NO$]
53	10.60	551.3289	300.1725	HMDB06768	19-Oxoandrost-4-ene-3,17-dione	$C_{19}H_{24}O_3$	$[M+H]^+$	-9	100.07569 [$C_5H_{10}NO$]
			300.1725	HMDB06772	Adrenosterone	$C_{19}H_{24}O_3$	$[M+H]^+$	-9	100.07569 [$C_5H_{10}NO$]
			300.1725	HMDB11195	2-Hydroxy-3-methoxyestrone	$C_{19}H_{24}O_3$	$[M+H]^+$	-9	100.07569 [$C_5H_{10}NO$]
54	10.61	637.3858	368.2199	HMDB03247	20-Hydroxy-PGE2	$C_{20}H_{32}O_6$	$[M+H+H_2O]^+$	-10	100.07569 [$C_5H_{10}NO$]
55*	10.74	379.2810	128.1201	HMDB01140	Octanal	$C_8H_{16}O$	$[M+H]^+$	-2	100.07569 [$C_5H_{10}NO$]
56	10.94	735.4283	466.2567	HMDB02829	Androsterone glucuronide	$C_{25}H_{38}O_8$	$[M+H+H_2O]^+$	-1	100.07569 [$C_5H_{10}NO$]
			466.2567	HMDB04484	Etiocholanolone glucuronide	$C_{25}H_{38}O_8$	$[M+H+H_2O]^+$	-1	100.07569 [$C_5H_{10}NO$]
			466.2567	HMDB06203	5-alpha-Dihydrotestosterone glucuronide	$C_{25}H_{38}O_8$	$[M+H+H_2O]^+$	-1	100.07569 [$C_5H_{10}NO$]
			466.2567	HMDB10365	3-alpha-hydroxy-5-alpha-androstane-17-one 3-D-glucuronide	$C_{25}H_{38}O_8$	$[M+H+H_2O]^+$	-1	100.07569 [$C_5H_{10}NO$]
57	10.99	505.2941	298.1205	HMDB04808	7C-aglycone	$C_{18}H_{18}O_4$	$[M+H-CO_2]^+$	3	100.07569 [$C_5H_{10}NO$]
58	11.03	483.1576	213.9879	HMDB06801	2-Oxo-3-hydroxy-4-phosphobutanoic acid	$C_4H_7O_8P$	$[M+H+H_2O]^+$	-5	100.07569 [$C_5H_{10}NO$]

59	11.06	635.3867	366.2042	HMDB06059	20-Carboxyleukotriene B4	$C_{20}H_{30}O_6$	$[M+H+H_2O]^+$	17	100.07569 [$C_5H_{10}NO$]
60	11.06	555.3301	286.1569	HMDB05895	4-Hydroxyestrone	$C_{18}H_{22}O_3$	$[M+H+H_2O]^+$	2	100.07569 [$C_5H_{10}NO$]
61	11.08	839.4876	606.3404	HMDB10361	(23S)-23,25-dihdroxy-24-oxovitamine D3 23-(beta-glucuronide)	$C_{33}H_{50}O_{10}$	$[M+H-H_2O]^+$	-4	100.07569 [$C_5H_{10}NO$]
62	11.66	393.2954	186.1256	HMDB10724	3-Oxodecanoic acid	$C_{10}H_{18}O_3$	$[M+H-CO_2]^+$	-4	100.07569 [$C_5H_{10}NO$]
63*	11.67	393.2952	142.1358	HMDB59835	Nonanal	$C_9H_{18}O$	$[M+H]^+$	-5	100.07569 [$C_5H_{10}NO$]
64	11.67	507.2986	239.1077	HMDB01510	Bupropion	$C_{13}H_{18}ClNO$	$[M+NH_4]^+$	6	100.07569 [$C_5H_{10}NO$]
65*	12.58	407.3099	156.1514	HMDB11623	Decanal	$C_{10}H_{20}O$	$[M+H]^+$	-7	100.07569 [$C_5H_{10}NO$]
66	13.60	438.1882	169.9980	HMDB01473	Dihydroxyacetone phosphate	$C_3H_7O_6P$	$[M+NH_4]^+$	5	100.07569 [$C_5H_{10}NO$]
67*	14.45	435.3412	184.1827	HMDB33933	Dodecanal	$C_{12}H_{24}O$	$[M+H]^+$	-7	100.07569 [$C_5H_{10}NO$]
68	15.54	586.2440	318.0641	HMDB04068	Melanin	$C_{18}H_{10}N_2O_4$	$[M+NH_4]^+$	-14	100.07569 [$C_5H_{10}NO$]
69	19.99	523.3838	290.2246	HMDB00031	Androsterone	$C_{19}H_{30}O_2$	$[M+H-H_2O]^+$	15	100.07569 [$C_5H_{10}NO$]
			290.2246	HMDB00365	Epiandrosterone	$C_{19}H_{30}O_2$	$[M+H-H_2O]^+$	15	100.07569 [$C_5H_{10}NO$]
			290.2246	HMDB00490	Etiocholanolone	$C_{19}H_{30}O_2$	$[M+H-H_2O]^+$	15	100.07569 [$C_5H_{10}NO$]
			290.2246	HMDB00546	Epietiocholanolone	$C_{19}H_{30}O_2$	$[M+H-H_2O]^+$	15	100.07569 [$C_5H_{10}NO$]
			290.2246	HMDB02961	Dihydrotestosterone	$C_{19}H_{30}O_2$	$[M+H-H_2O]^+$	15	100.07569 [$C_5H_{10}NO$]
			272.2140	HMDB06046	5a-Androst-3-en-17-one	$C_{19}H_{28}O$	$[M+H]^+$	16	100.07569 [$C_5H_{10}NO$]
			290.2246	HMDB06770	5b-Dihydrotestosterone	$C_{19}H_{30}O_2$	$[M+H-H_2O]^+$	15	100.07569 [$C_5H_{10}NO$]
70	19.99	479.2494	210.0740	HMDB03219	Sedoheptulose	$C_7H_{14}O_7$	$[M+H+H_2O]^+$	7	100.07569 [$C_5H_{10}NO$]
71	20.06	721.5091	470.3396	HMDB11628	Glycyrrhetinic acid	$C_{30}H_{46}O_4$	$[M+H]^+$	11	100.07569 [$C_5H_{10}NO$]
72	21.13	507.3636	270.2195	HMDB10733	3-Oxohexadecanoic acid	$C_{16}H_{30}O_3$	$[M+H-CH_2]^+$	-3	100.07569 [$C_5H_{10}NO$]
73	21.15	809.5845	576.4179	HMDB06820	2-Hexaprenyl-3-methyl-5-hydroxy-6-methoxy-1,4-benzoquinone	$C_{38}H_{56}O_4$	$[M+H-H_2O]^+$	19	100.07569 [$C_5H_{10}NO$]
74	21.53	566.4306	298.2508	HMDB10736	3-Oxooctadecanoic acid	$C_{18}H_{34}O_3$	$[M+NH_4]^+$	-15	100.07569 [$C_5H_{10}NO$]
75	21.56	671.5270	402.3498	HMDB06892	7a-Hydroxy-5b-cholestan-3-one	$C_{27}H_{46}O_2$	$[M+H+H_2O]^+$	8	100.07569 [$C_5H_{10}NO$]
76	22.08	682.5507	414.3498	HMDB12113	(22Alpha)-hydroxy-campest-4-en-3-one	$C_{28}H_{46}O_2$	$[M+NH_4]^+$	19	100.07569 [$C_5H_{10}NO$]

Supplementary Figures

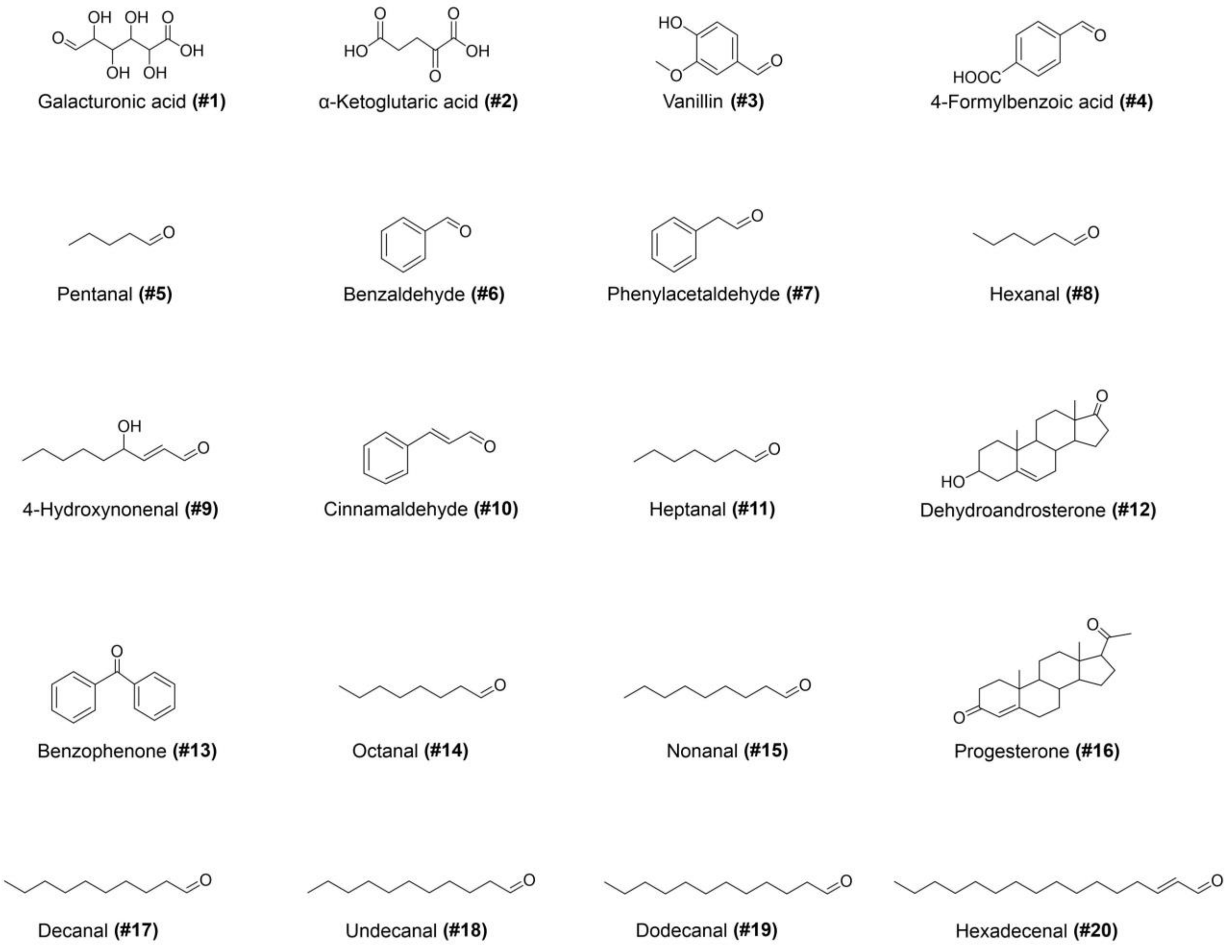

Figure S1. The chemical structures of 20 carbonyl standards.

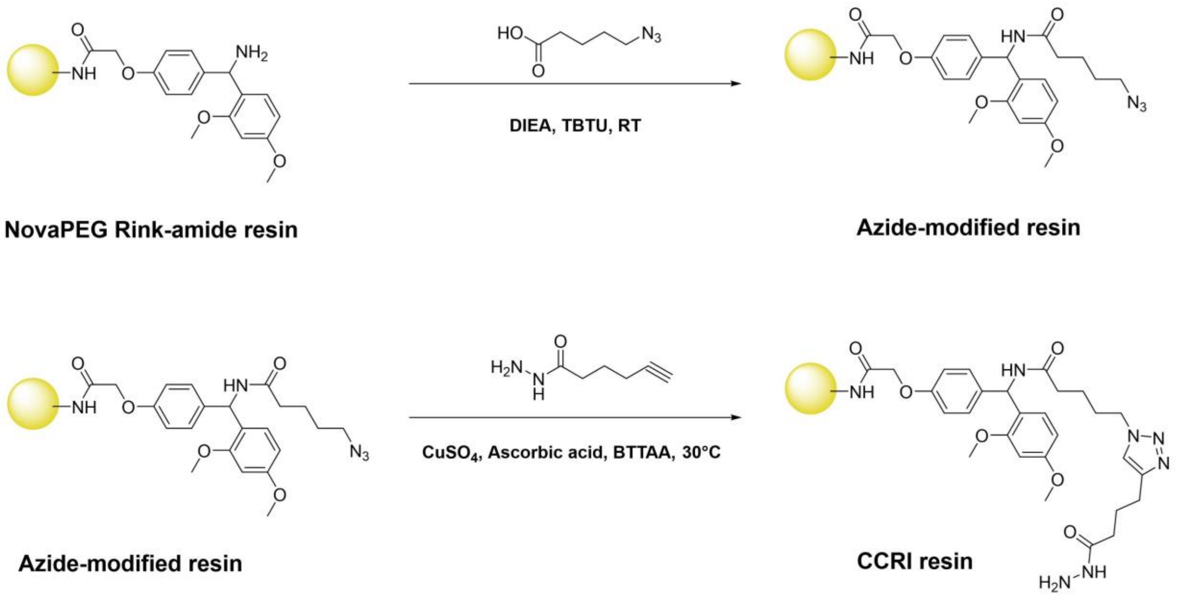

Figure S2. The synthesis of CCRI resins using a two-step process involving acylation reaction and click chemistry. DIEA, N,N-diisopropylethylamine; TBTU, 2-(1H-benzotriazole-1-yl)-1,1,3,3-tetramethylammonium tetrafluoroborate; RT, room temperature, BTTAA, 2-(4-((Bis((1-(tert-butyl)-1H-1,2,3-triazol-4-yl)methyl)amino)methyl)-1H-1,2,3-triazol-1-yl) acetic acid.

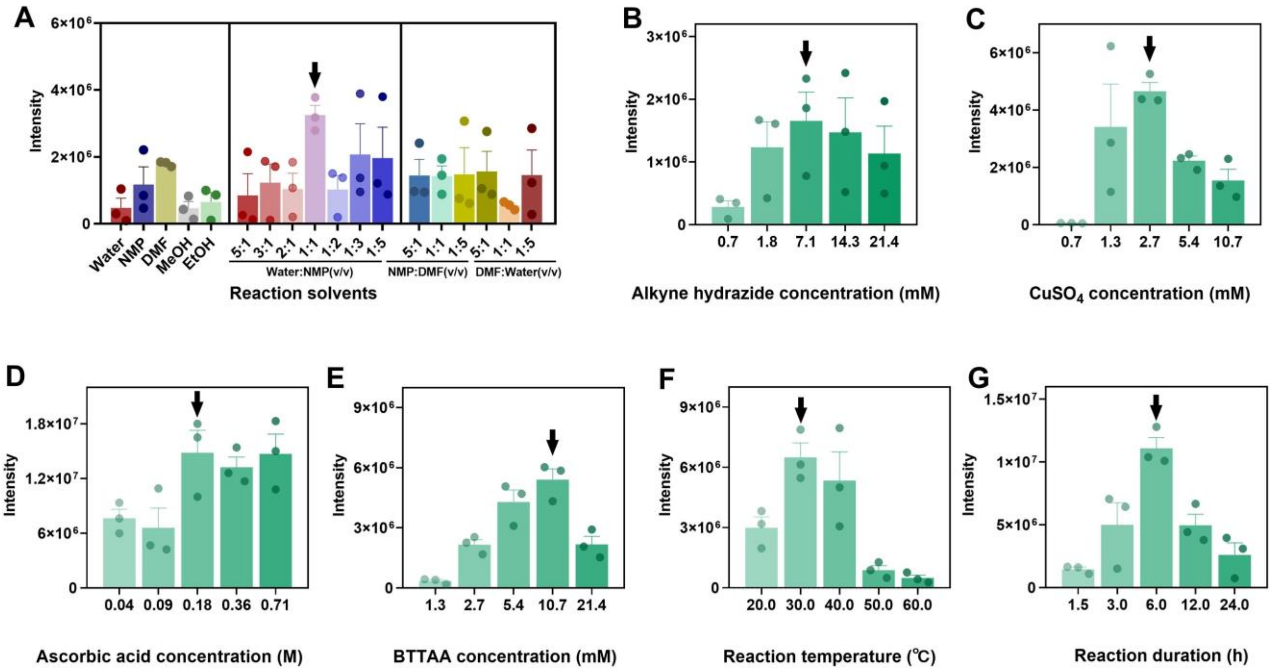

Figure S3. Optimization of Cu(I)-catalyzedazide-alkyne cycloaddition (CuAAC) reaction conditions for the synthesis of CCRI resin. (**A**) Reaction solvents, (**B**) alkyne hydrazide concentration, (**C**) $CuSO_4$ concentration, (**D**) ascorbic acid concentration, (**E**) BTTAA concentration, (**F**) reaction temperature, and (**G**) reaction duration.

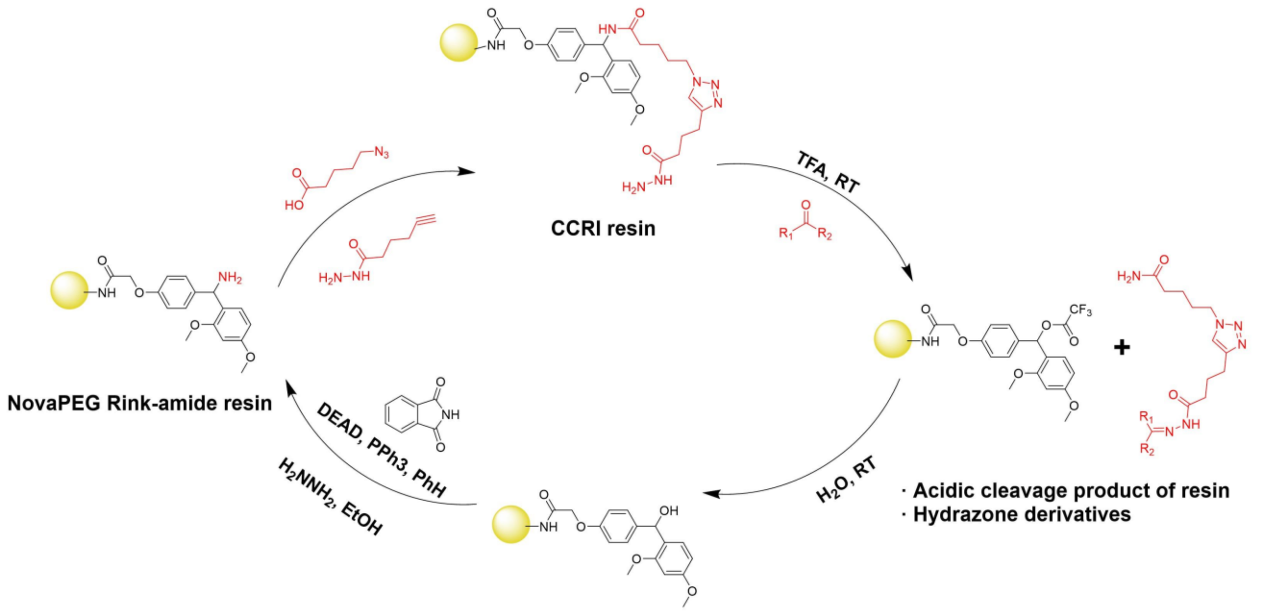

Figure S4. The regeneration of the immobilized CCRI resin. TFA, Trifluoroacetic acid; RT, Room temperature; DEAD, Diethyl azodicarboxylate; PPh3, Triphenylphosphine

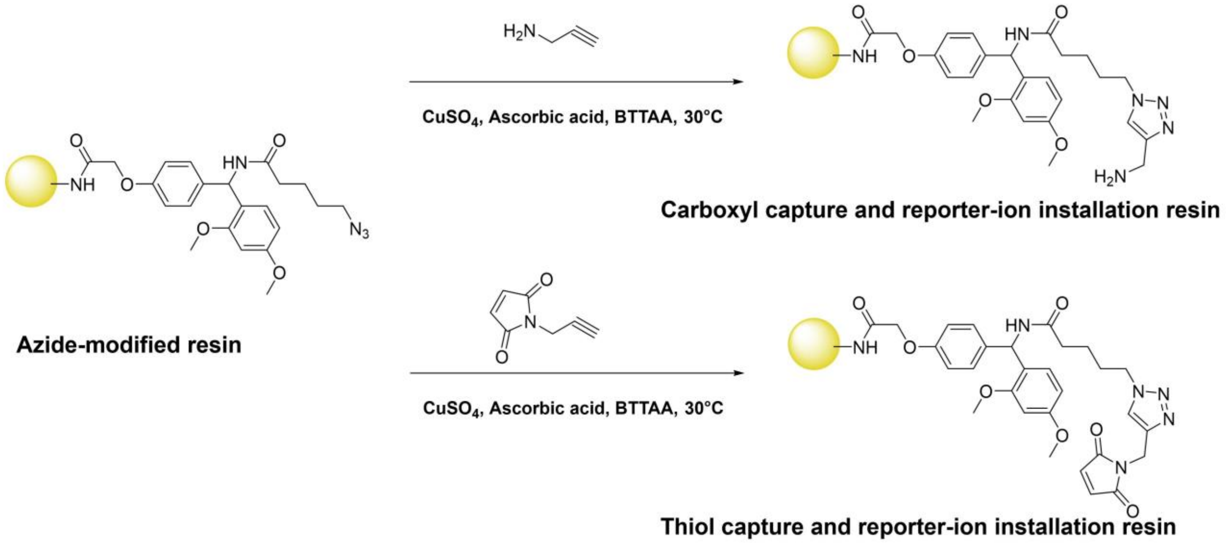

Figure S5. The synthesis of carboxyl or thiol capture and reporter-ion installation resins.

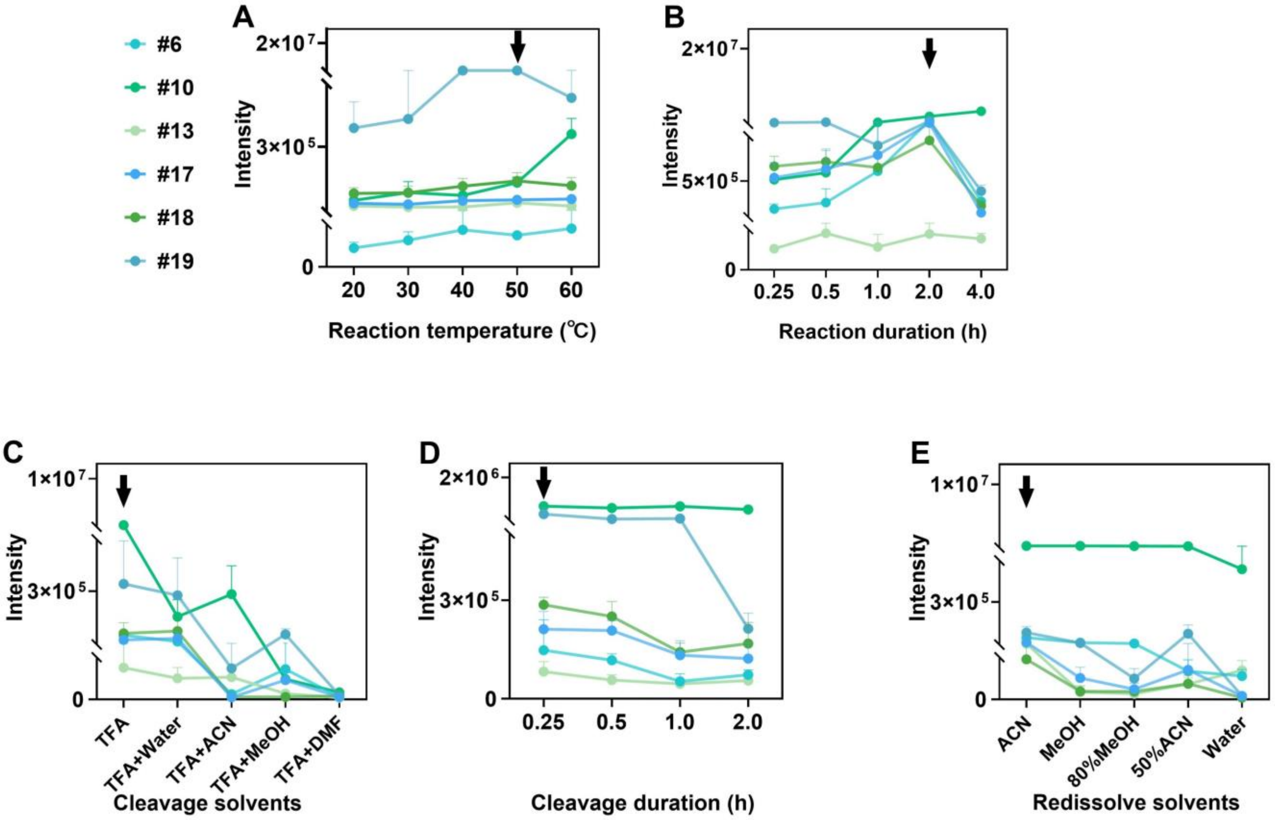

Figure S6. Optimization of Girard derivatization conditions, cleavage reaction conditions and redissolve solvent using 6 representative carbonyl standards. (**A**) Reaction temperature, and (**B**) reaction duration for Girard derivatization; (**C**) cleavage solvents, (**D**) cleavage duration, and (**E**) redissolve solvents. The numbers of carbonyl standards are represented in the same manner as in **Table 1**.

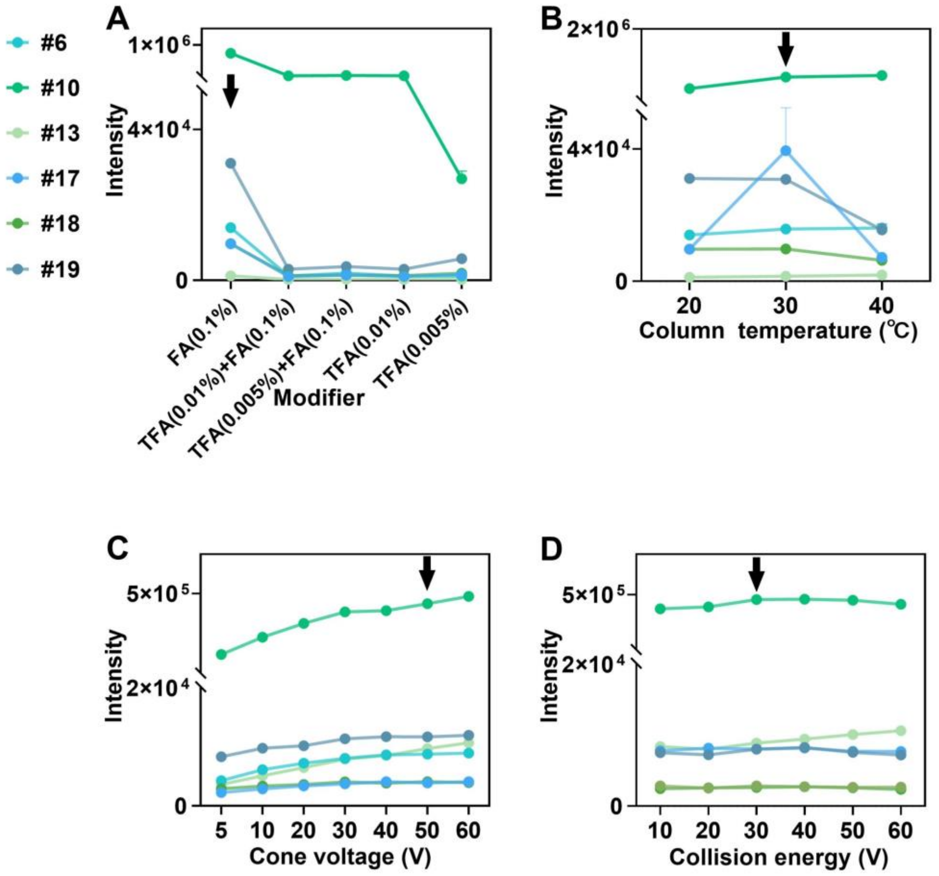

Figure S7. Optimization of LC-MS conditions using 6 representative carbonyl standards. (**A**) Mobile phase modifiers, (**B**) column temperature, (**C**) cone voltage, and (**D**) collision energy. The numbers of carbonyl standards are represented in the same manner as in **Table 1**.

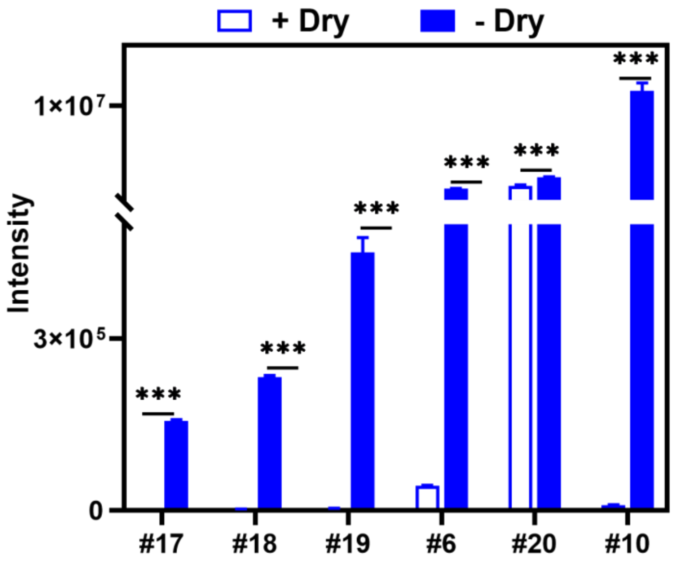

Figure S8. Effects of drying step during sample processing on the signal of carbonyl metabolites using GDBE method. The numbers of carbonyl standards are represented in the same manner as in **Table 1**

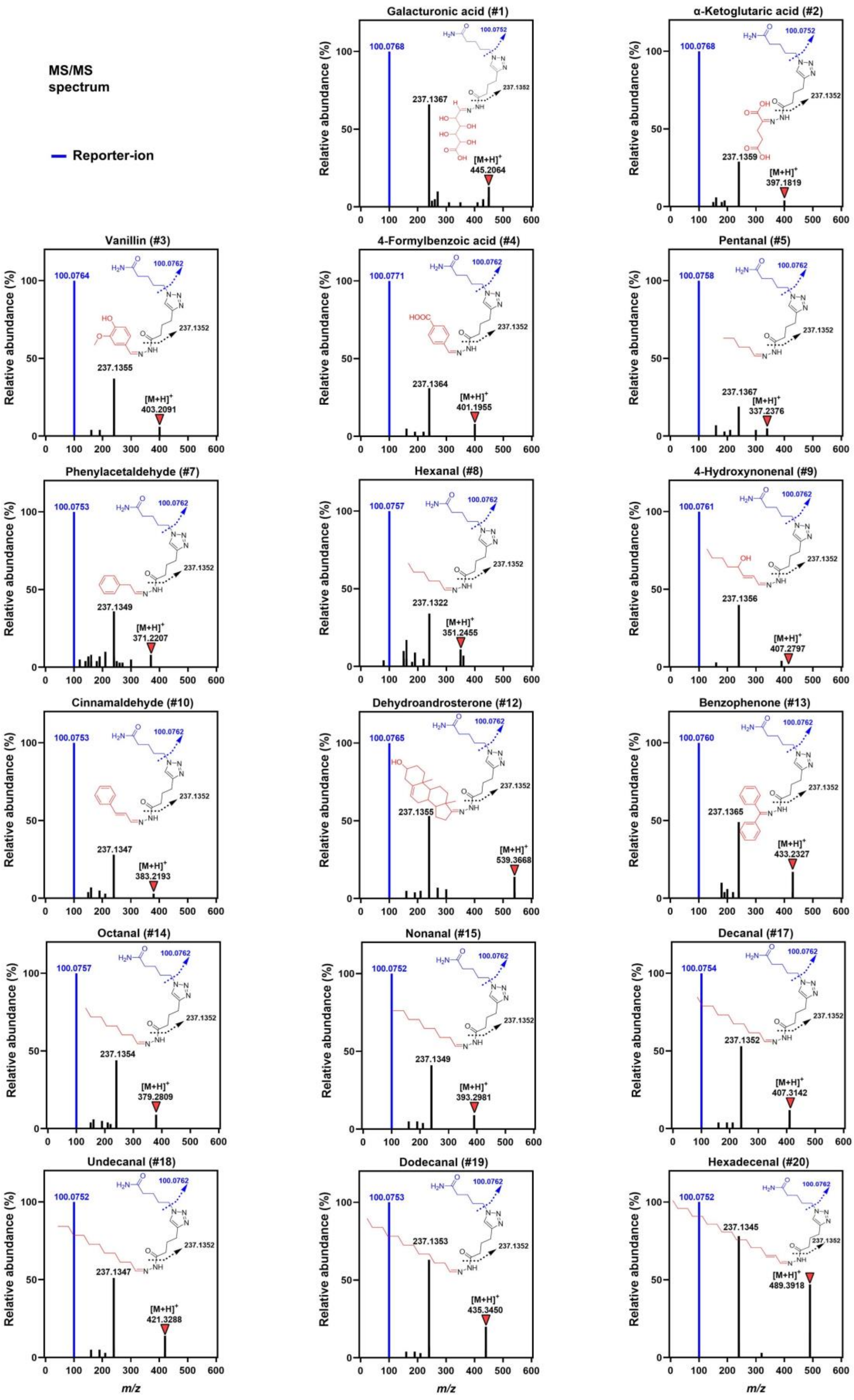

Figure S9. MS/MS spectra and fragmentation patterns of carbonyl metabolites in the form of hydrazone derivative detected in the positive ion mode.

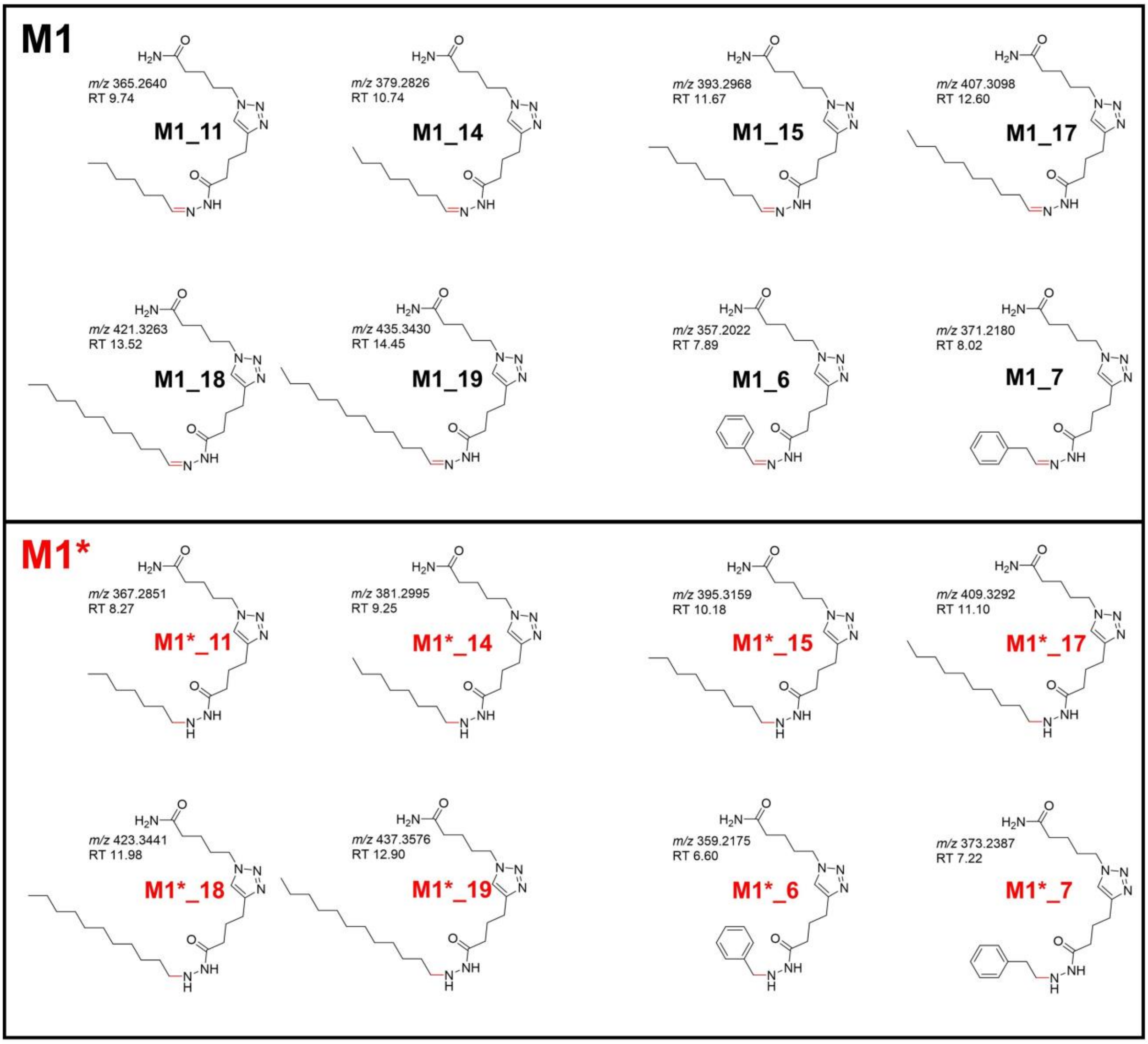

Figure S10. The chemical structures of the hydrazone derivatives derived from carbonyl metabolites without a C=C bond (M1) and their possible reduction products (M1*).

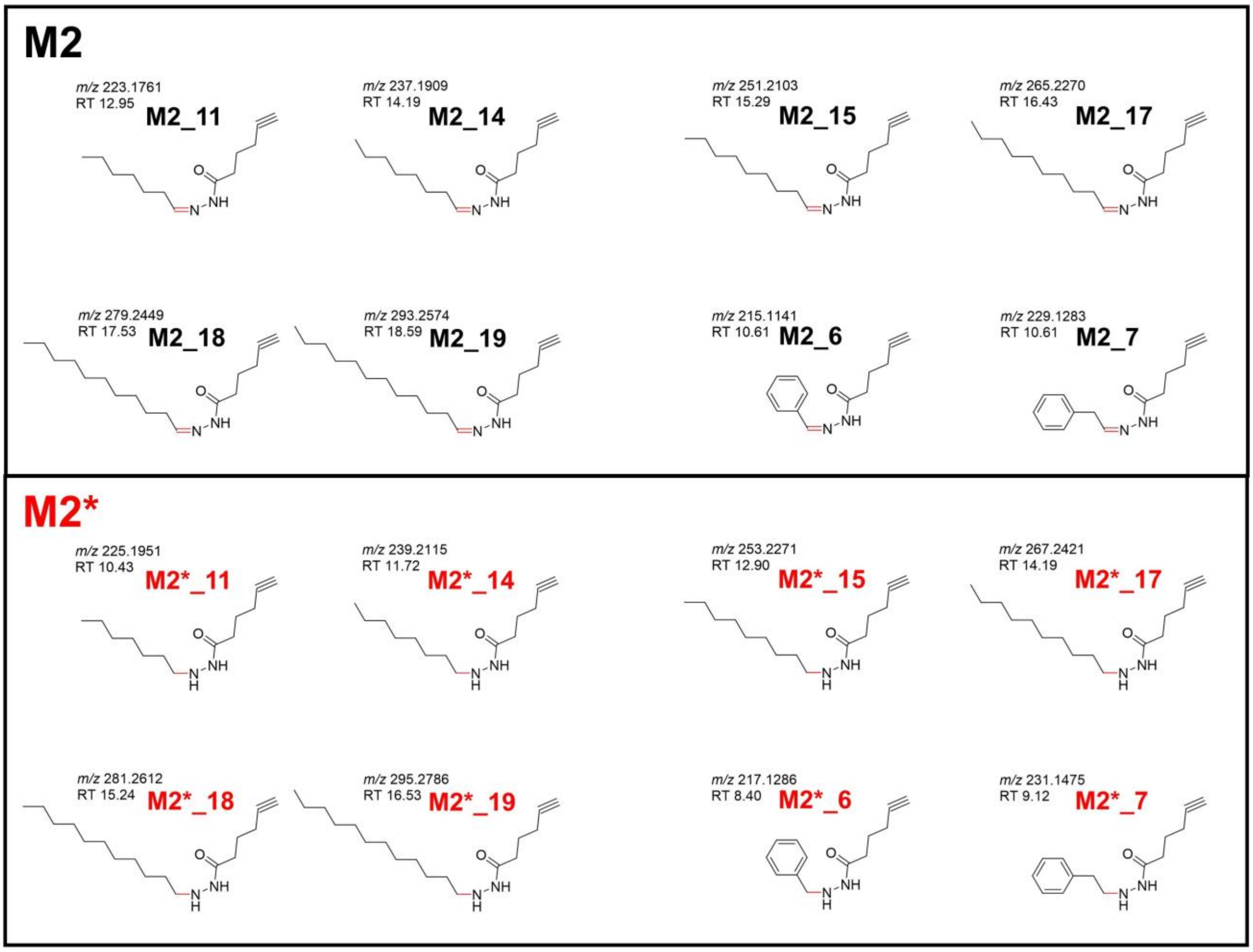

Figure S11. The chemical structures of alkyne hydrazide-derivatized carbonyls (M2) and their possible reduction products (M2*).

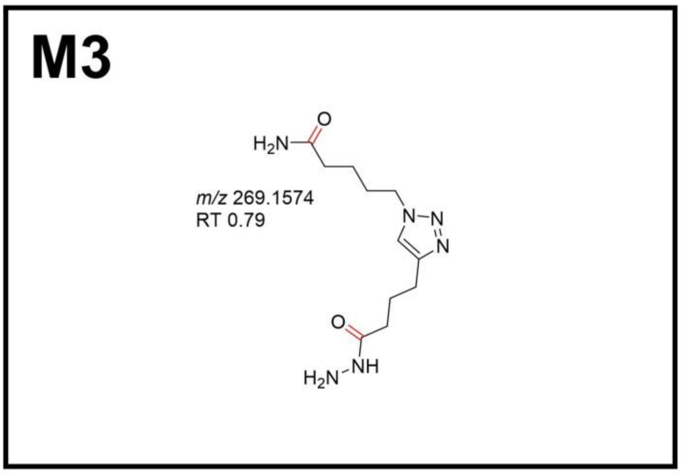

Figure S12. The chemical structures of the cleaved products of CCRI resin.

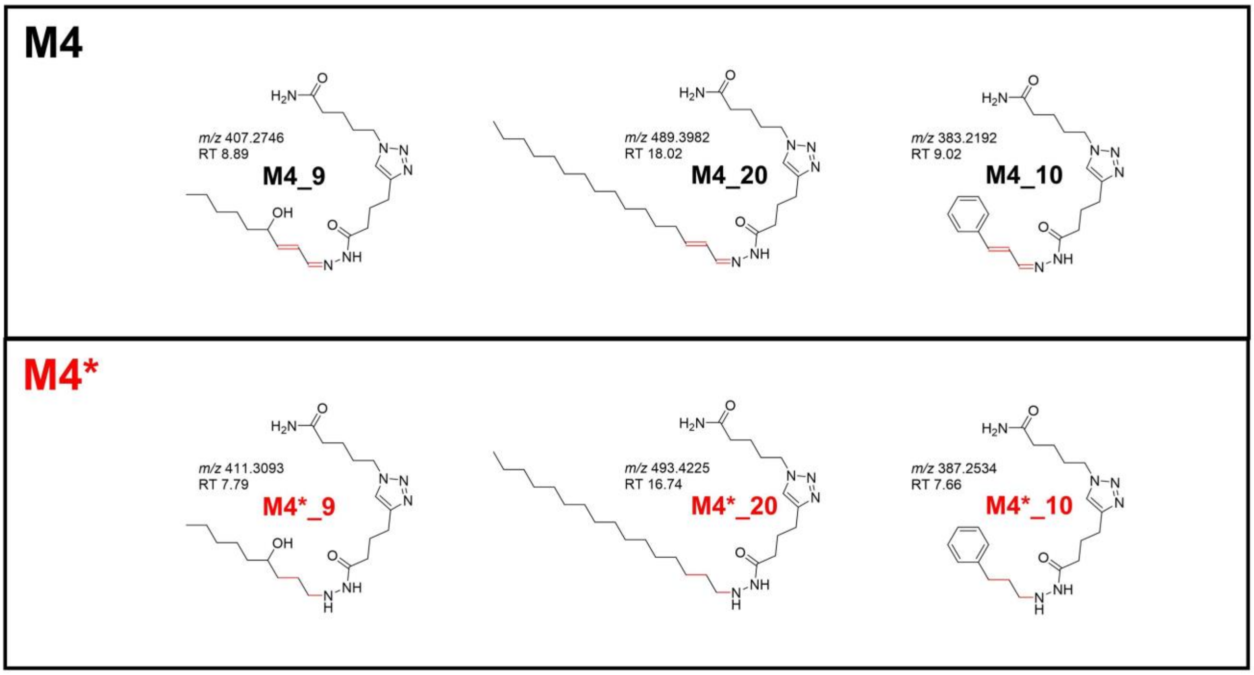

Figure S13. The chemical structures of the expected hydrazone derivatives derived from carbonyl metabolites with a conjugated C=C bond (M4) and their possible reduction products (M4*).

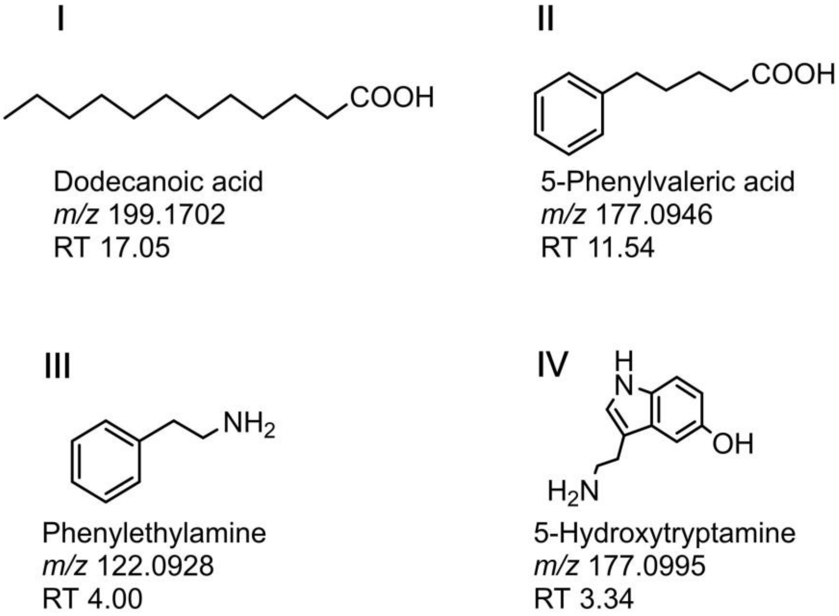

Figure S14. Chemical structures of endogenous metabolite standards without carbonyl group, including carboxylic acids (I and II) and amines (III and IV) in the selectivity experiments.

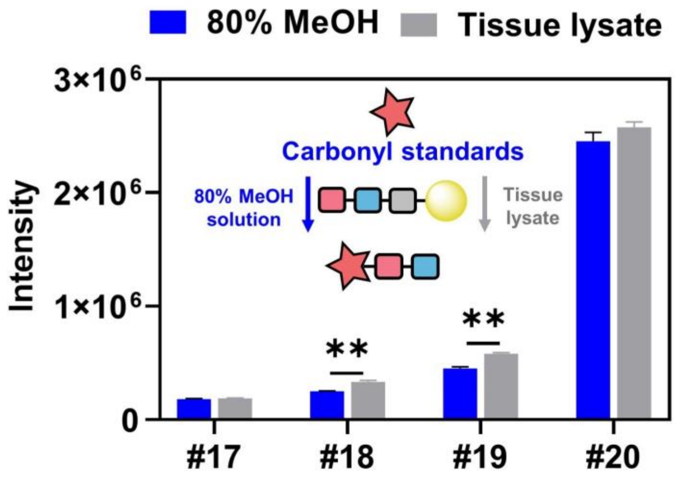

Figure S15. The influence of GDBE on matrix effects. Mixed carbonyl standards were added to either an 80% methanol solution or liver tissue lysate, and subjected to CCRI resin labeling. The numbers of carbonyl standards are represented in the same manner as in **Table 1**.

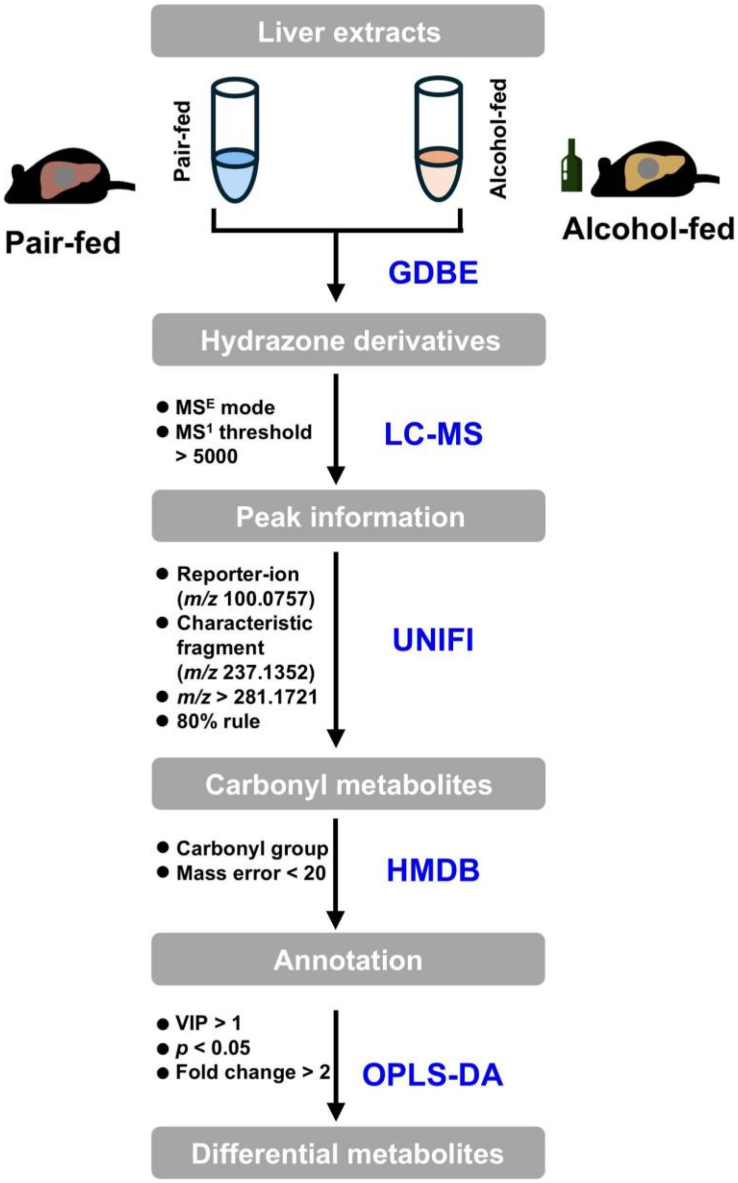

Figure S16. The workflow of comprehensive profiling and relative quantification for the discovery and annotation of carbonyl metabolites from mice liver using GDBE strategy.
